# Mice lacking **γ**ENaC palmitoylation sites maintain benzamil-sensitive Na^+^ transport despite reduced channel activity

**DOI:** 10.1172/jci.insight.172051

**Published:** 2023-11-08

**Authors:** Andrew J. Nickerson, Stephanie M. Mutchler, Shaohu Sheng, Natalie A. Cox, Evan C. Ray, Ossama B. Kashlan, Marcelo D. Carattino, Allison L. Marciszyn, Aaliyah Winfrey, Sebastien Gingras, Annet Kirabo, Rebecca P. Hughey, Thomas R. Kleyman

**Affiliations:** 1 Department of Medicine,; 2 Department of Computational and Systems Biology,; 3 Department of Cell Biology, and; 4 Department of Immunology, University of Pittsburgh, Pittsburgh, Pennsylvania, USA.; 5 Department of Medicine, Vanderbilt University Medical Center, Nashville, Tennessee, USA.; 6 Department of Pharmacology and Chemical Biology, University of Pittsburgh, Pittsburgh, Pennsylvania, USA.

**Keywords:** Cell Biology, Nephrology, Epithelial transport of ions and water, Ion channels

## Abstract

Epithelial Na^+^ channels (ENaCs) control extracellular fluid volume by facilitating Na^+^ absorption across transporting epithelia. In vitro studies showed that Cys-palmitoylation of the γENaC subunit is a major regulator of channel activity. We tested whether γ subunit palmitoylation sites are necessary for channel function in vivo by generating mice lacking the palmitoylated cysteines (γ^C33A,C41A^) using CRISPR/Cas9 technology. ENaCs in dissected kidney tubules from γ^C33A,C41A^ mice had reduced open probability compared with wild-type (WT) littermates maintained on either standard or Na^+^-deficient diets. Male mutant mice also had higher aldosterone levels than WT littermates following Na^+^ restriction. However, γ^C33A,C41A^ mice did not have reduced amiloride-sensitive Na^+^ currents in the distal colon or benzamil-induced natriuresis compared to WT mice. We identified a second, larger conductance cation channel in the distal nephron with biophysical properties distinct from ENaC. The activity of this channel was higher in Na^+^-restricted γ^C33A,C41A^ versus WT mice and was blocked by benzamil, providing a possible compensatory mechanism for reduced prototypic ENaC function. We conclude that γ subunit palmitoylation sites are required for prototypic ENaC activity in vivo but are not necessary for amiloride/benzamil-sensitive Na^+^ transport in the distal nephron or colon.

## Introduction

Na^+^ transport in the aldosterone-sensitive distal nephron (ASDN) is a primary determinant of extracellular fluid volume and blood pressure (1, 2). Epithelial Na^+^ channel–mediated (ENaC-mediated) transport in principal cells of the ASDN is one of the most highly regulated renal Na^+^ handling mechanisms (3–7), controlled mainly by volume-regulatory hormones such as aldosterone (8), vasopressin (9), and angiotensin II (10). ENaC activity also facilitates K^+^ secretion in the ASDN by providing the depolarizing force necessary for K^+^ efflux into the tubule lumen (11). Specific loss-of-function ENaC mutations cause type 1 pseudo-hypoaldosteronism (PHA-1), a disorder of renal salt wasting and hyperkalemia (12–14). Gain-of-function mutations have been described in patients with Liddle syndrome, where reduced rates of ENaC degradation lead to early-onset hypertension and hypokalemia (15, 16). Other ENaC gain-of-function variants may also contribute to salt-sensitive hypertension (17–22).

ENaCs are heterotrimeric (αβγ), amiloride-sensitive channels that are regulated by several mechanisms (23). Synthesis and apical membrane trafficking of channel subunits are both aldosterone-regulated events that increase the number of channels (*N*) at the cell surface (24, 25). Other factors that increase ENaC open probability (*P_O_*) include proteolytic cleavage of the α and γ subunits (26–28), binding of acidic phospholipids such as phosphatidylinositol 4,5-bisphosphate (29–31), extracellular Na^+^ and H^+^ (32–34), shear stress (35), and Cys-palmitoylation (36–38).

Cys-palmitoylation is the reversible attachment of a 16-carbon acyl group (palmitate) to intracellular cysteines. ENaC β and γ subunits were shown to be palmitoylated, which increases the channel’s *P_O_* (36, 37). Palmitoylation of the γ subunit at 2 residues in the intracellular N-terminus (γC33 and γC41) was necessary for normal channel activity in vitro (37). Channels lacking either palmitoylation site had substantially reduced *P_O_* but no decrease in surface expression of α or γ subunits. Coexpression with a palmitoyltransferase (DHHC2) enhances currents from wild-type (WT) but not palmitoylation site–deficient channels (γ^C33A,C41A^) expressed in *Xenopus* oocytes. Accordingly, catalytically dead DHHC2 did not increase WT currents. Inhibition of endogenous palmitoyltransferase activity using 2-bromopalmitate reduces amiloride-sensitive ENaC currents in cultured mouse cortical collecting duct cells (37). Specific DHHC proteins co-immunoprecipitate with ENaC and increase currents in cultured cells, and at least 1 isoform (DHHC3) colocalizes with ENaC in mouse kidney (38). However, the physiological importance of ENaC regulation by palmitoylation in vivo remains unknown.

**Figure 1:**
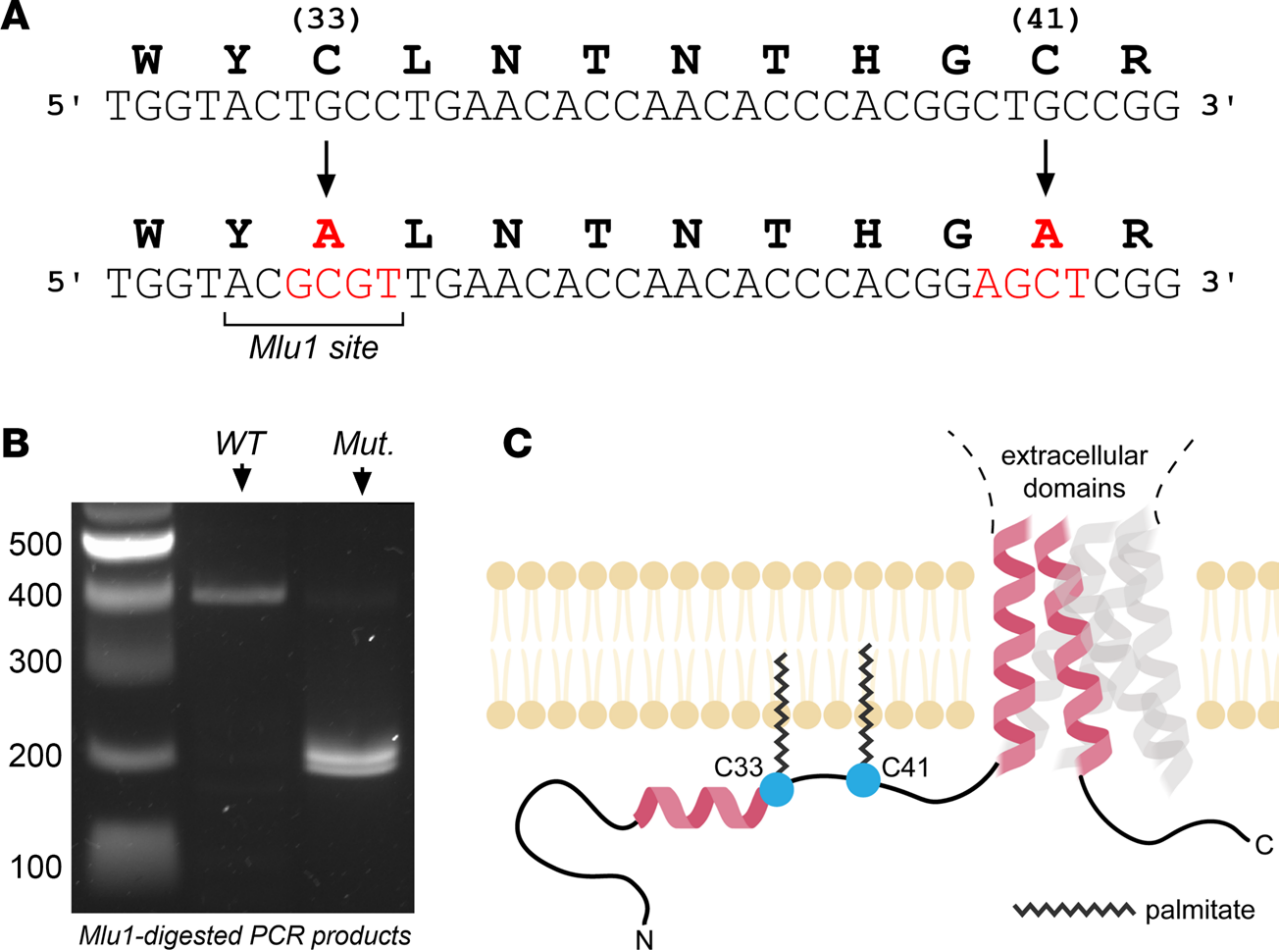
Schematic for the γ^C33A,C41A^ mouse genotype. (**A**) Amino acid (upper) and nucleotide (lower) partial sequences of mouse γ subunit’s N-terminal region. CRISPR-mediated cysteine to alanine mutations are shown in red. (**B**) Genotyping of WT and γ^C33A,C41A^ mice, showing PCR products after digestion with *Mlul1* (see Methods for details). DNA ladder and corresponding molecular weights are shown to the left. (**C**) Cartoon depicting the locations of putative palmitoylation sites, C33 and C41, within γ subunit’s N-terminal region (α helices within the γ subunit are depicted in red; α and β subunits are translucent).

We developed a mouse model lacking γ subunit palmitoylation sites (γ^C33A,C41A^) using CRISPR/Cas9 technology to study the role of this mechanism in regulating ENaC in vivo (Figure 1). Using electrophysiological approaches, we assessed ENaC function in the kidneys and distal colons of WT and γ^C33A,C41A^ littermates under normal and Na^+^-depleted conditions. We also assessed ENaC subunit expression and processing, as well as systemic electrolyte handling via metabolic cage analyses. Our results demonstrate that γ subunit palmitoylation sites, γC33 and γC41, are required for channel activity in vivo. However, salt and fluid balance can still be maintained, likely because of other benzamil-sensitive transport mechanisms.

## Results

### γ^C33A,C41A^ mice express ENaCs with reduced activity in the cortical connecting tubules/collecting ducts but maintain normal blood electrolytes and basal metabolic parameters.

**Figure 2:**
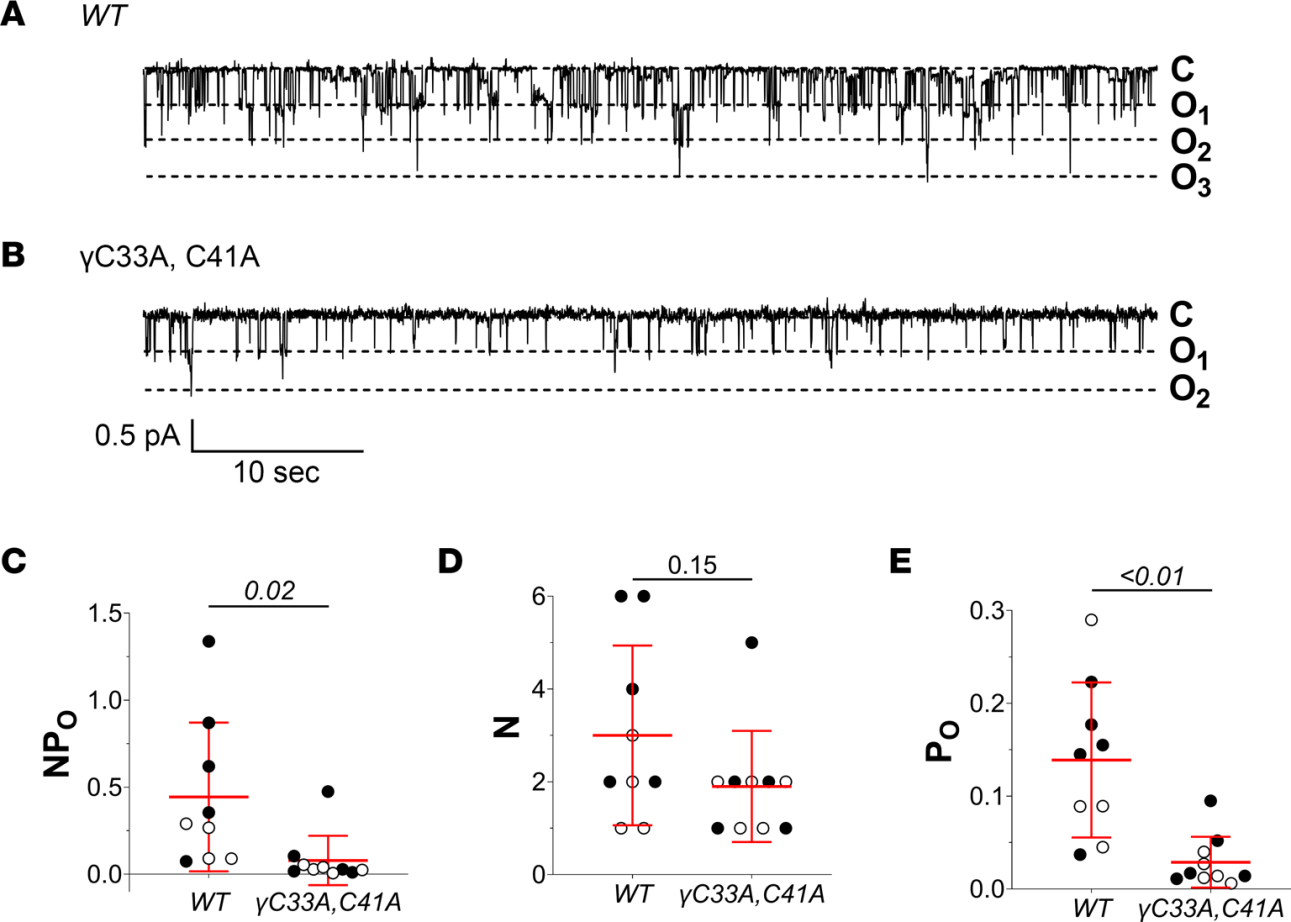
Baseline ENaC activity is reduced in split-open cortical connecting tubules /collecting ducts (CNT/CCDs) from γ^C33A,C41A^ versus WT mice. (**A** and **B**) Representative recordings from cell-attached patches of principal cell (PC) apical membranes in split-open CNT/CCDs from WT (**A**) and γ^C33A,C41A^ (**B**) mice maintained on a standard chow diet. Channel closed (C) and open (O) states are depicted to the right of each recording. (**C**–**E**) Total channel activity (*NP_O_*; **C**), number of observed channels per patch (*N*; **D**), and single-channel open probability (*P_O_*: **E**) in apical membrane patches from WT (*n* = 9) and γ^C33A,C41A^ mice (*n* = 10). Data from male and female mice are shown as black and white circles, respectively. Lines and error bars represent mean ± SD. *NP_O_*, *N*, and *P_O_* data were analyzed via Student’s unpaired *t* test.

We first assessed whether γ^C33A,C41A^ mice exhibited phenotypic abnormalities at baseline due to possible deficits in ENaC-dependent Na^+^ absorption. Age-matched γ^C33A,C41A^ mice and WT littermate controls (6–7 weeks) showed no differences in blood electrolytes or plasma aldosterone levels when maintained on a standard chow diet (Table 1). However, cell-attached patch clamp recordings from split-open cortical connecting tubules/collecting ducts (CNT/CCDs) revealed that γ^C33A,C41A^ mice expressed ENaCs with significantly reduced activity at baseline compared with WT controls. This was detected as a decrease in total channel activity (*NP_O_*; measured as number of channels × *P_O_*), driven by a reduced *P_O_* without a change in the number of observed channels per patch (Figure 2, C–E). The very low apparent single-channel *P_O_* from γ^C33A,C41A^ mice (Figure 2E) reduced the likelihood of all channels opening simultaneously, leading to a likely underestimation of *N* and an overestimation of *P_O_*. An obvious reduction in *NP_O_* was nonetheless apparent, indicating a decrease in total ENaC activity in the apical membranes of PCs from γ^C33A,C41A^ compared with WT mice. This observation is consistent with published data from heterologous expression systems using channels that carry the same γ subunit cysteine mutations (38). Estimated single-channel conductance was similar between genotypes (WT vs. γ^C33A,C41A^: 7.3 ± 0.9 vs. 8.2 ± 0.7 pS with Li^+^ as the charge carrier; *P* = 0.11).

### ENaC protein expression is not altered in γ^C33A,C41A^ versus WT mouse kidney.

**Figure 3:**
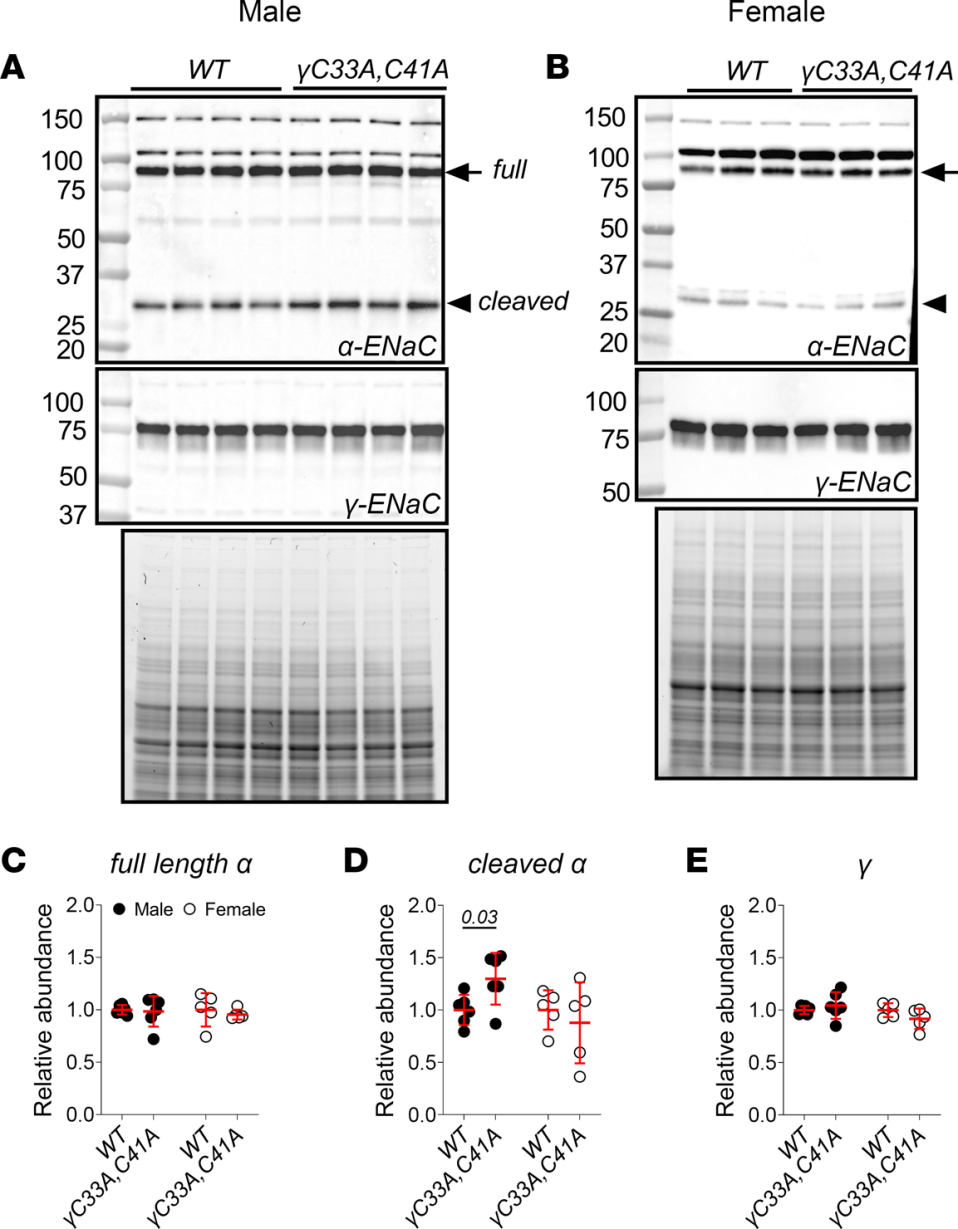
Baseline ENaC α and γ subunit expression is similar between WT and γ^C33A,C41A^ mice. (**A** and **B**) Representative Western blot images showing the detection of ENaC subunits in kidney homogenates from male (**A**) and female (**B**) WT and γ^C33A,C41A^ mice maintained on a standard chow diet. Standard molecular weight markers are shown in the far-left lane, along with corresponding weights, in kDa. Full-length α subunit, as well as the cleaved N-terminal fragment, are indicated by arrows and arrowheads, respectively. Stain-free gel images showing total protein content for each sample are shown below blots. (**C**–**E**) Densitometric quantification of full-length α subunit (**C**), cleaved α subunit (**D**), and γ subunit (**E**) was performed by first normalizing to the total protein signal for each sample, then transforming the data such that WT values were set equal to 1 (WT and γ^C33A,C41A^ males, *n* = 6; WT and γ^C33A,C41A^ females, *n* = 5). Lines and error bars represent mean ± SD. Comparisons were made via Student’s unpaired *t* test for each sex.

Under basal conditions, we did not observe a change in either α or γ subunit expression between WT and γ^C33A,C41A^ mouse kidney homogenates (Figure 3; see complete unedited blots in the supplemental material). These results again comport with previous findings in Fischer rat thyroid cells or *Xenopus* oocytes. We did detect a small increase in the N-terminal cleavage fragment of the α subunit in γ^C33A,C41A^ versus WT male mice (Figure 3D) but not female mice. These data hint that male mice may be more sensitive to the observed loss of ENaC function.

### ENaC activity in the CNT/CCD remains low in γ^C33A,C41A^ mice under dietary Na^+^ restriction.

ENaC-mediated absorption in the ASDN has a minor role in Na^+^ and fluid homeostasis in mice under basal dietary conditions. However, during dietary Na^+^ restriction, elevated aldosterone levels dramatically enhance ENaC activity, which then becomes a more prominent component to tubular Na^+^ reabsorption (8, 25, 39). We therefore evaluated the effects of Na^+^ restriction on channel activity and systemic salt and fluid balance in these mice.

**Figure 4:**
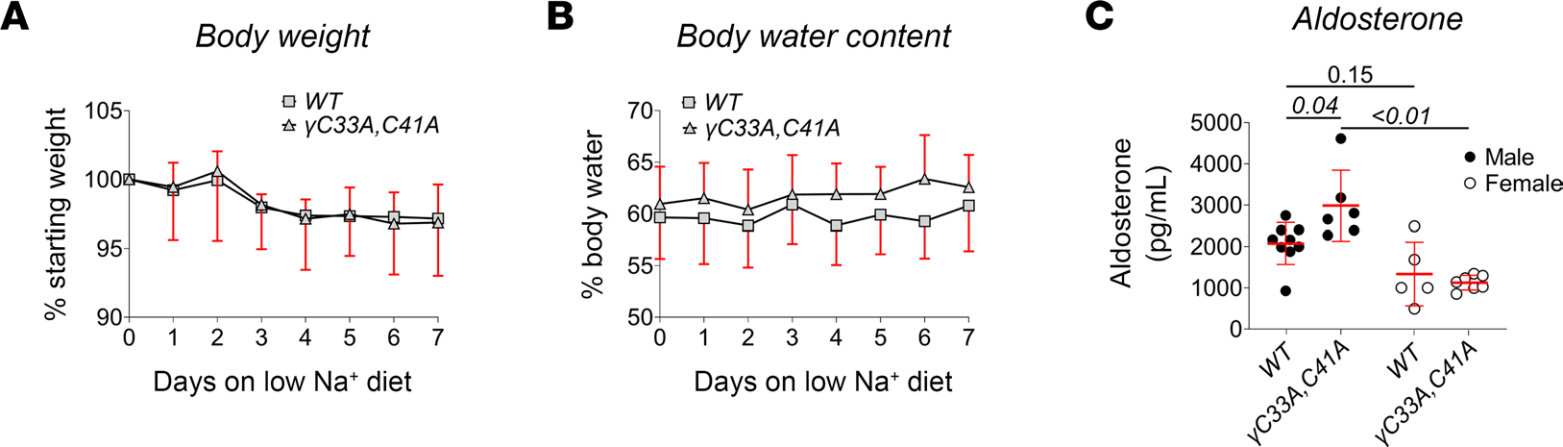
Dietary Na^+^ restriction induces higher aldosterone levels in male γ^C33A,C41A^ compared with male WT mice. (**A** and **B**) Body weight (**A**) and total body water percentage (**B**) measured in WT (squares) and ENaCγ^C33A,C41A^ mice (triangles) of both sexes during 7 consecutive days on low-Na^+^ (<0.01%) diet (*n* = 9 for WT, *n* = 11 γ^C33A,C41A^). (**C**) Plasma aldosterone levels measured in WT versus γ^C33A,C41A^ mice after 8 days of dietary Na^+^ depletion (*n* = 9 for WT male, *n* = 6 for γ^C33A,C41A^ male, *n* = 5 for WT female, *n* = 7 for γ^C33A,C41A^ female). Data from male and female mice are shown as black and white circles, respectively. Lines and error bars represent mean ± SD. Time course percentage starting body weight and percentage body water measurements were compared via 2-way ANOVA with repeated measures. No significant differences were found regarding body weight or water content between the groups. Endpoint aldosterone levels were assessed via 2-way ANOVA and multiple comparisons made with Tukey’s post hoc analysis.

During 7 days on a low-Na^+^ diet (<0.01%), mutant mice maintained similar body weight and total body water composition compared to WT littermates (Figure 4, A and B), suggesting no global deficits in maintaining fluid balance. Blood electrolytes and metabolic parameters were also similar between groups following treatment (Table 2). However, plasma aldosterone levels were significantly higher in male γ^C33A,C41A^ mice than in WT male controls (Figure 4C and Table 2), indicating these animals were more sensitive to Na^+^ restriction and required more aldosterone to maintain homeostasis. As with α subunit cleavage on a Na^+^-replete diet (Figure 3), this effect was not observed in females. Sex differences with respect to salt handling in the nephron have been reported (40) and are likely involved in the differing compensatory responses between male and females in this experiment.

**Figure 5:**
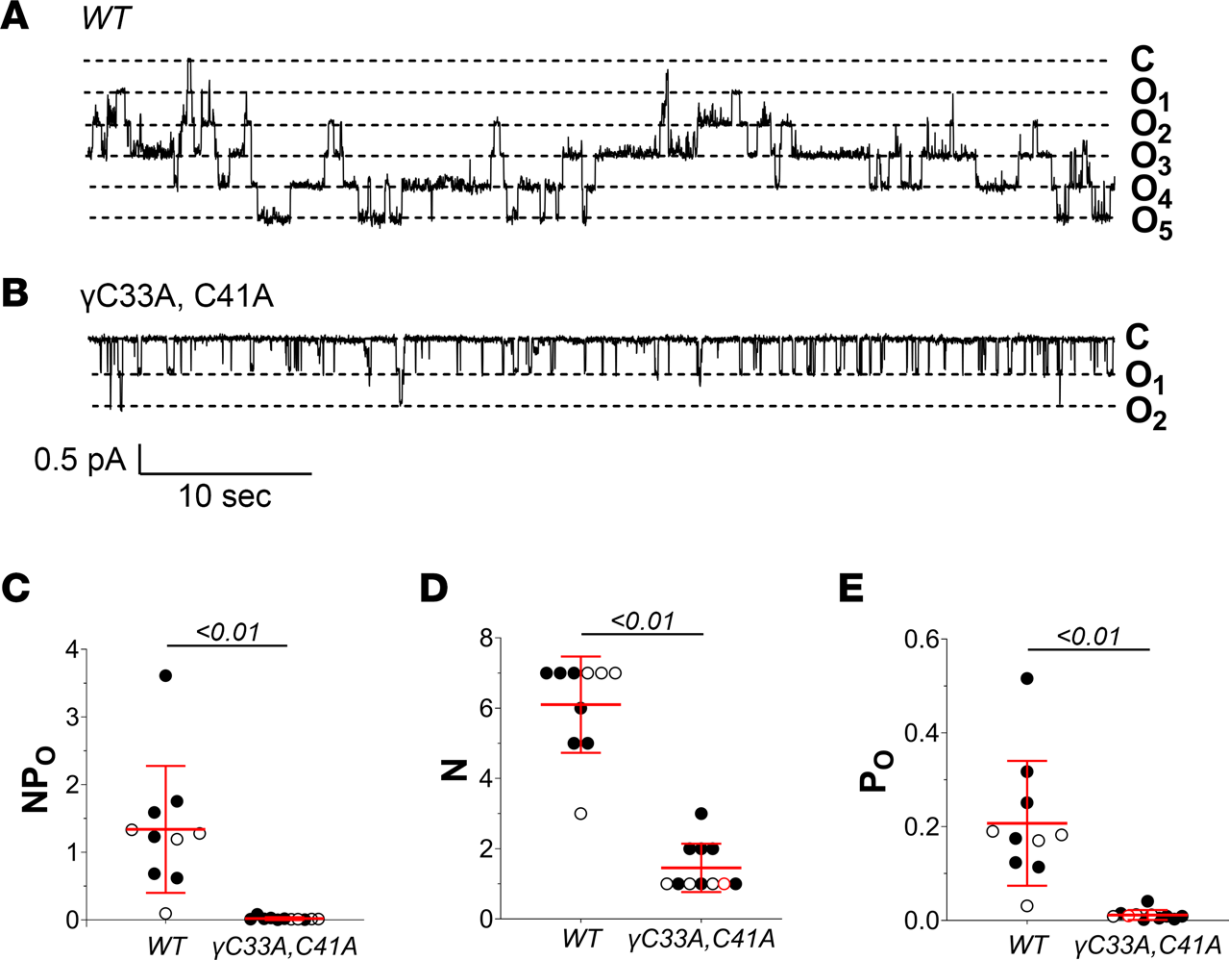
CNT/CCD ENaC activity in γ^C33A,C41A^ mice remains low under dietary Na^+^ restriction. (**A** and **B**) Representative recordings from cell-attached patches of PC apical membranes in split-open CNT/CCDs from WT (**A**) and γ^C33A,C41A^ mice (**B**) maintained on a low-Na^+^ (<0.01%) diet. Channel closed (C) and open (O) states are depicted to the right of each recording. (**C**–**E**) Total channel activity (*NP_O_*; **C**), number of observed channels per patch (*N*; **D**), and single-channel open probability (*P_O_*: **E**) in apical membrane patches from WT (*n* = 10) and γ^C33A,C41A^ (*n* = 11) mouse collecting ducts. Data from male and female mice are shown as black and white circles, respectively. Lines and error bars represent mean ± SD. *NP_O_*, *N*, and *P_O_* data were analyzed via Student’s unpaired *t* test.

Cell-attached recordings of PC apical membranes from WT mice maintained on low-Na^+^ diet revealed robust ENaC activity, as expected (Figure 5A). Under these conditions, *NP_O_* was approximately 3 times higher compared with WT mice on a standard diet (1.3 ± 0.9 vs. 0.4 ± 0.4, *P* = 0.02 by unpaired *t* test; Figure 2C vs. Figure 5C). However, channel activity in γ^C33A,C41A^ mice remained very low, even after Na^+^ restriction (Figure 5, C–E). This suggests that channels lacking γ subunit N-terminal cysteines cannot be activated by elevated aldosterone signaling that is associated with dietary Na^+^ restriction. As mentioned above, the very low *P_O_* observed in mutant mice likely results in an underestimation of *N* in these experiments.

**Figure 6:**
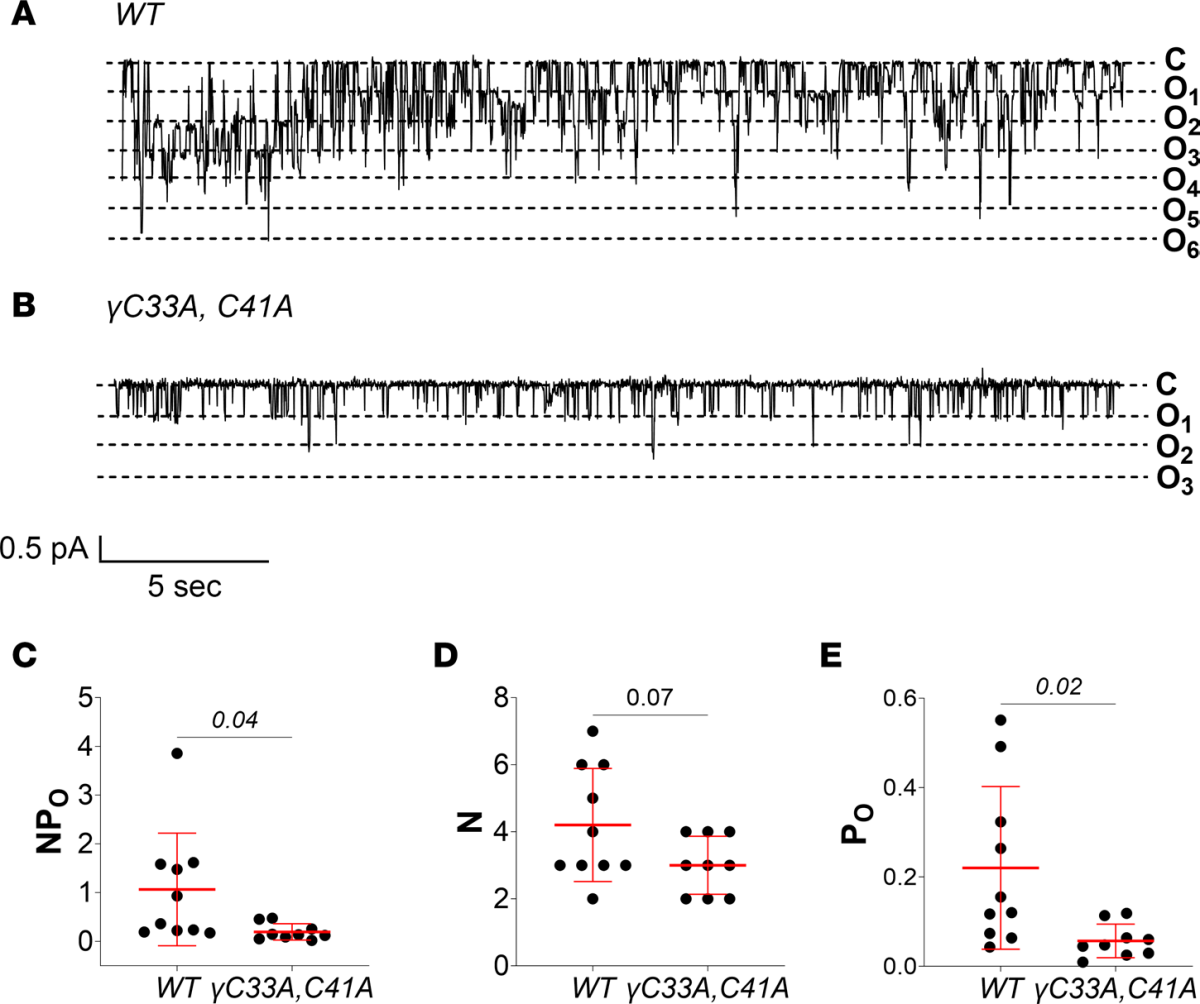
ENaC activity in the DCT2/CNT is reduced in γ^C33A,C41A^ mice under dietary Na^+^ restriction. (**A** and **B**) Representative recordings from cell-attached patches of DCT2/CNT cell apical membranes from male WT (**A**) and γ^C33A,C41A^ mice (**B**) maintained on a low-Na^+^ (<0.01%) diet. Channel closed (C) and open (O) states are depicted to the right of each recording. (**C**–**E**) Total channel activity (*NP_O_*; **C**), number of observed channels per patch (*N*; **D**), and single-channel open probability (*P_O_*; **E**) in apical membrane patches from WT (*n* = 10) and γ^C33A,C41A^ (*n* = 9) mouse DCT2/CNTs. Lines and error bars represent mean ± SD. *NP_O_*, *N*, and *P_O_* data were analyzed via Student’s unpaired *t* test.

ENaC activity is also observed in the late distal convoluted tubule (DCT2) and early CNT (8). In the DCT2, relatively low levels of 11β-hydroxysteroid dehydrogenase expression permit mineralocorticoid receptor activation by glucocorticoids (i.e., corticosterone/cortisol), rather than mineralocorticoids (i.e., aldosterone) (41). ENaC activity in the DCT2/CNT is therefore robust under both normal and Na^+^-restricted conditions. Whether ENaC activation by palmitoylation is region specific within the ASDN is unclear. Because ENaC regulation differs between the DCT2 and more distal segments, we assessed whether augmented channel activity in the DCT2/CNT might compensate for the loss of function observed in CNT/CCDs from γ^C33A,C41A^ mice. Cell-attached recordings revealed that ENaC activity in the DCT2/CNT was also significantly reduced in γ^C33A,C41A^ versus WT mice (Figure 6). This indicates that ENaC function in γ^C33A,C41A^ mice is reduced throughout the ASDN and is not a region-specific phenomenon.

### WT and γ^C33A,C41A^ mice have comparable γ subunit cleavage.

One major consequence of aldosterone signaling in the distal nephron is ENaC γ subunit cleavage (42–44), which activates the channel by releasing a short inhibitory peptide located in the extracellular domain (28, 45). This event increases *P_O_*, in part, by rendering the channel insensitive to the inhibitory effect of extracellular Na^+^ (i.e., by removing Na^+^ self-inhibition) (46). We therefore investigated whether our mutation impaired γ subunit proteolysis in mice, as this could explain the reduced channel activity observed under high-aldosterone conditions.

**Figure 7:**
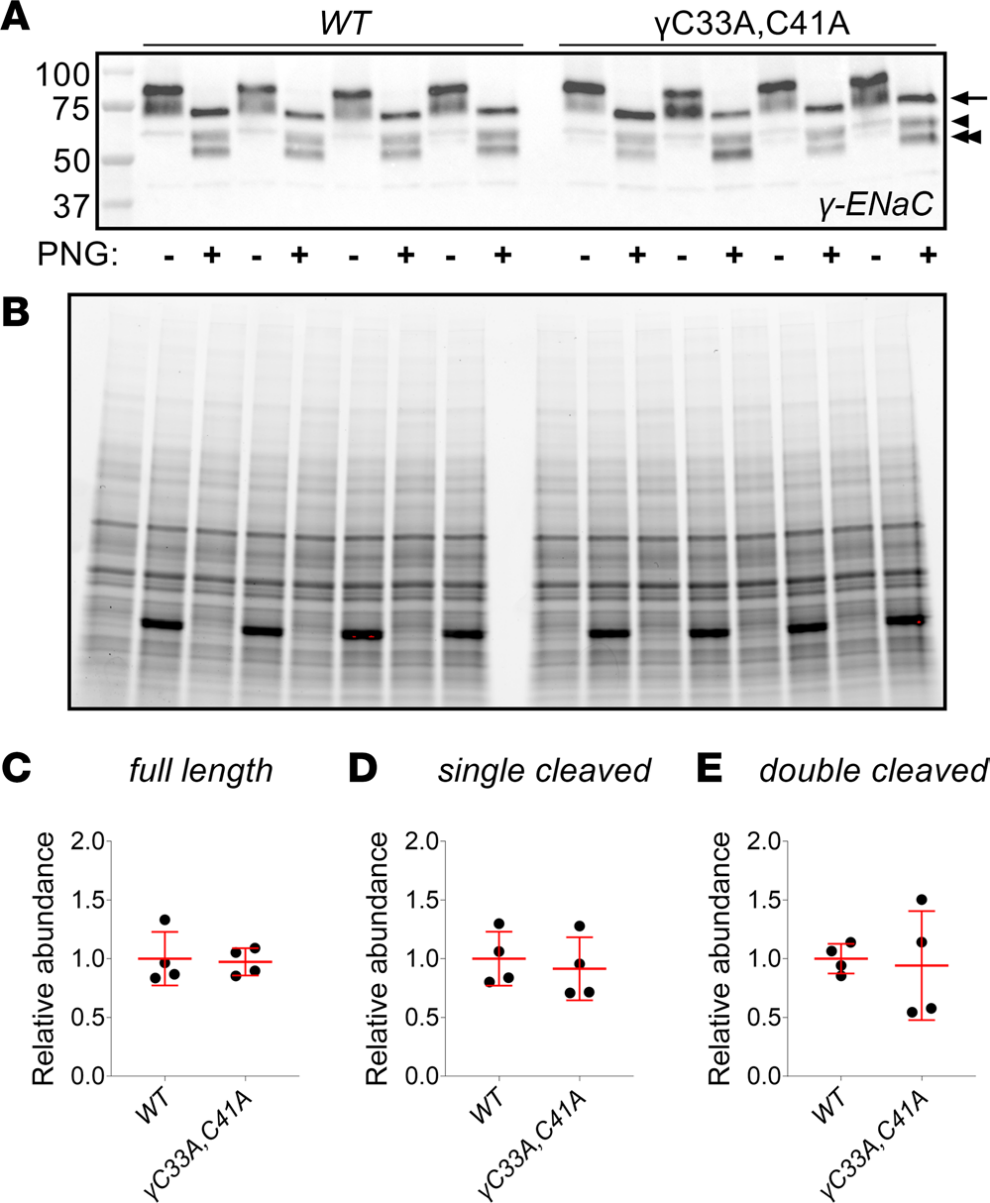
Expression and cleavage pattern of the γ subunit is similar between Na^+^-restricted WT and γ^C33A,C41A^ mice. (**A**) Western blot image showing cleavage fragments of the γ subunit after treating samples with (+) or without (-) PNGase F to remove N-linked sugar residues. Standard molecular weight markers are shown in the far-left lane, along with corresponding weights, in kDa. Arrow indicates the full, uncleaved fragment. Single and double arrowheads indicate proximally (furin) and distally (presumably double) cleaved fragments, respectively. All samples were from male mice maintained for 8 days on a low-Na^+^ diet. (**B**) Stain-free gel image showing total protein content for each sample. Dark bands appearing in the stain-free gel image correspond to PNGase enzyme (~36 kDa) in the sample preparation. Densitometric quantification of full-length (**C**) and furin (**D**) distally cleaved γ subunit (**E**) was performed as described above (*n* = 4 for WT and γ^C33A,C41A^ mice). Lines and error bars represent mean ± SD. Statistical comparisons were made via Student’s unpaired *t* test.

We examined γ subunit cleavage by treating kidney lysates with peptide:N-glycosidase F (PNGase F) to deglycosylate the protein prior to SDS-PAGE, as previously described (47). This approach facilitates visualization of the uncleaved subunit protein (~70 kDa), subunits processed at the proximal furin cleavage site (~55 kDa), and subunits processed by other proteases at a distal cleavage site (~50 kDa). The 50 kDa band likely reflects twice-cleaved γ subunits corresponding to a fully activated form of the channel. Patterns of γ subunit cleavage were identical between WT and γ^C33A,C41A^ mice under Na^+^-restricted conditions, as were the overall levels of γ subunit expression (Figure 7). This suggests that the decreased ENaC activity observed in γ^C33A,C41A^ mice reflects a reduction in *P_O_* that is independent of channel proteolysis.

### WT and γ^C33A,C41A^ mice have similar expression of upstream Na^+^ transport proteins, NCC and NKCC2.

**Figure 8:**
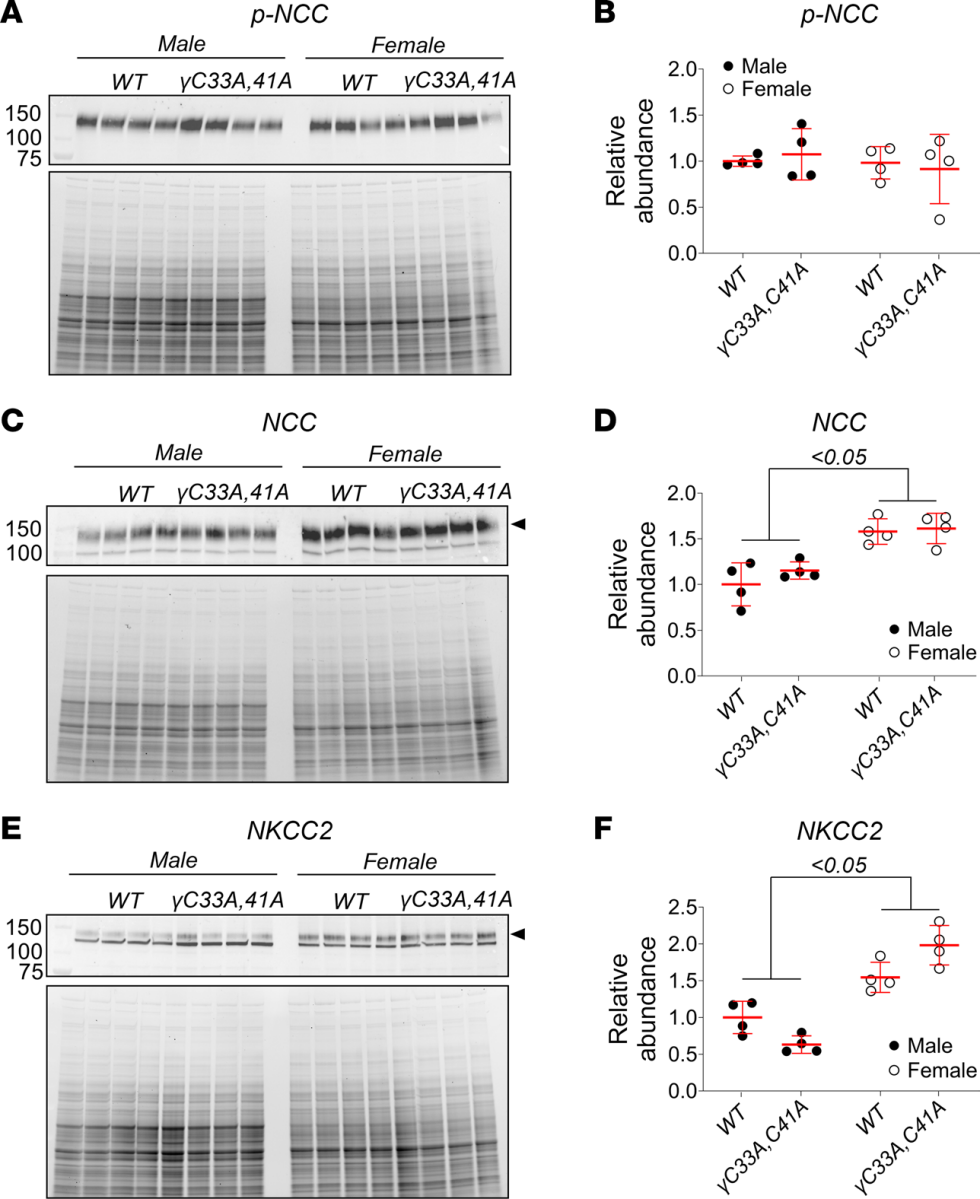
Expression of upstream Na^+^ transport proteins is similar between Na^+^-restricted WT and γ^C33A,C41A^ mice but varies by sex. (**A**, **C**, and **E**) Western blot images showing expression of Thr-53 phosphorylated Na^+^,Cl^–^ co-transporter (p-NCC; **A**), total NCC (**C**), and Na^+^, K^+^, 2Cl^–^ co-transporter (NKCC2; **E**) proteins from whole-kidney lysates of WT and γ^C33A,C41A^ mice of both sexes (*n* = 4 for each group). Data from male and female mice are shown as black and white circles, respectively. Standard molecular weight markers are shown in the far-left lane, along with corresponding weights, in kDa. Stain-free gel images showing total protein content for each sample are shown below each blot. All mice were maintained for 8 days on a low-Na^+^ diet. (**B**, **D**, and **F**) Densitometric quantification of p-NCC (**B**), total NCC (**D**), and NKCC2 (**E**) was performed as described above. Lines and error bars represent mean ± SD. Statistical comparisons were made via 2-way ANOVA with Tukey’s post hoc analysis.

Given that ENaC activity in cell-attached patch recordings was remarkably low in γ^C33A,C41A^ mice even under Na^+^-restricted conditions, we assessed whether Na^+^ transport mechanisms in other upstream nephron segments may be altered in order to compensate for loss of ENaC function. We measured the expression of 2 other critical Na^+^ transporters, NCC and NKCC2, which mediate electroneutral Na^+^ reabsorption in the distal convoluted tubule and thick ascending limb, respectively. Western blot analysis revealed no differences in the expression of either total NCC protein or its active phosphorylated form (phosphothreonine at position 53) between WT and γ^C33A,C41A^ mice of either sex (Figure 8, A–D). NKCC2 expression was also similar between WT and γ^C33A,C41A^ mice (Figure 8, E and F). These data indicate that γ^C33A,C41A^ mice do not require enhanced Na^+^ transport in upstream segments to maintain homeostasis, despite substantial loss of ENaC function.

Interestingly, NCC and NKCC2 co-transporters were both more highly expressed in female versus male mice, regardless of genotype (Figure 8, D and F). This suggests that females may rely more heavily on Na^+^ reabsorption in the distal convoluted tubule and/or the thick ascending limb under Na^+^-restricted conditions. This finding may also explain, at least in part, the lower aldosterone levels measured in female versus male mice after maintenance on a low-Na^+^ diet (Figure 4C).

### WT and γ^C33A,C41A^ mice display similar responses to chronic and acute K^+^ loading.

**Figure 9:**
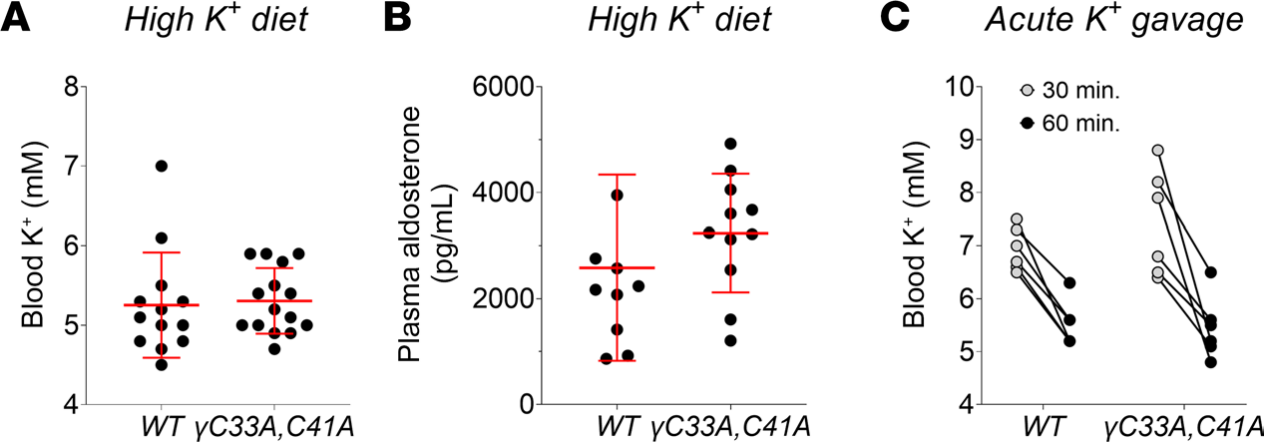
Responses of WT and γ^C33A,C41A^ mice to K^+^ loading. (**A** and **B**) Blood K^+^ (**A**) and plasma aldosterone levels (**B**) measured from WT and γ^C33A,C41A^ (*n* = 13 and 15, respectively for **A**; *n* = 10 and 11, respectively for **B**) male mice maintained on a 10% KCl diet for 8 days. Lines and error bars represent mean ± SD. Statistical comparisons were made via Student’s unpaired *t* test. (**C**) Blood K^+^ levels measured following an acute oral gavage of 150 μL 5% KCl solution in WT (*n* = 6) and γ^C33A,C41A^ (*n* = 6) mice maintained on a standard chow diet. Sequential measurements were taken at 30- and 60-minute time points postgavage. Statistical comparison was made with 2-way ANOVA with repeated measures. No difference between genotypes was observed.

ENaC is required for K^+^ excretion in the ASDN, although under certain conditions some degree of ENaC-independent K^+^ excretion has been reported (11). Clinical use of ENaC-selective blockers (e.g., amiloride) is limited for this reason, as treatment often causes hyperkalemia (48). Given that channel activity was quite low in γ^C33A,C41A^ mice, we tested whether this conferred increased sensitivity to K^+^ loading. To this end, we administered both chronic (10% KCl diet for 8 days) and acute (150 μL gavage of 5% KCl) K^+^ loads. In either case, we did not detect increased blood K^+^ for γ^C33A,C41A^ compared to WT mice (Figure 9, A and C). Aldosterone levels after chronic K^+^ loading were also similar between the groups (Figure 9B). Thus, if there was any impairment in the ability of γ^C33A,C41A^ mice to deal with an excess K^+^ load, the effect was too subtle to detect.

### WT and ENaCγ^C33A,C41A^ mice display robust benzamil-sensitive natriuresis.

**Figure 10:**
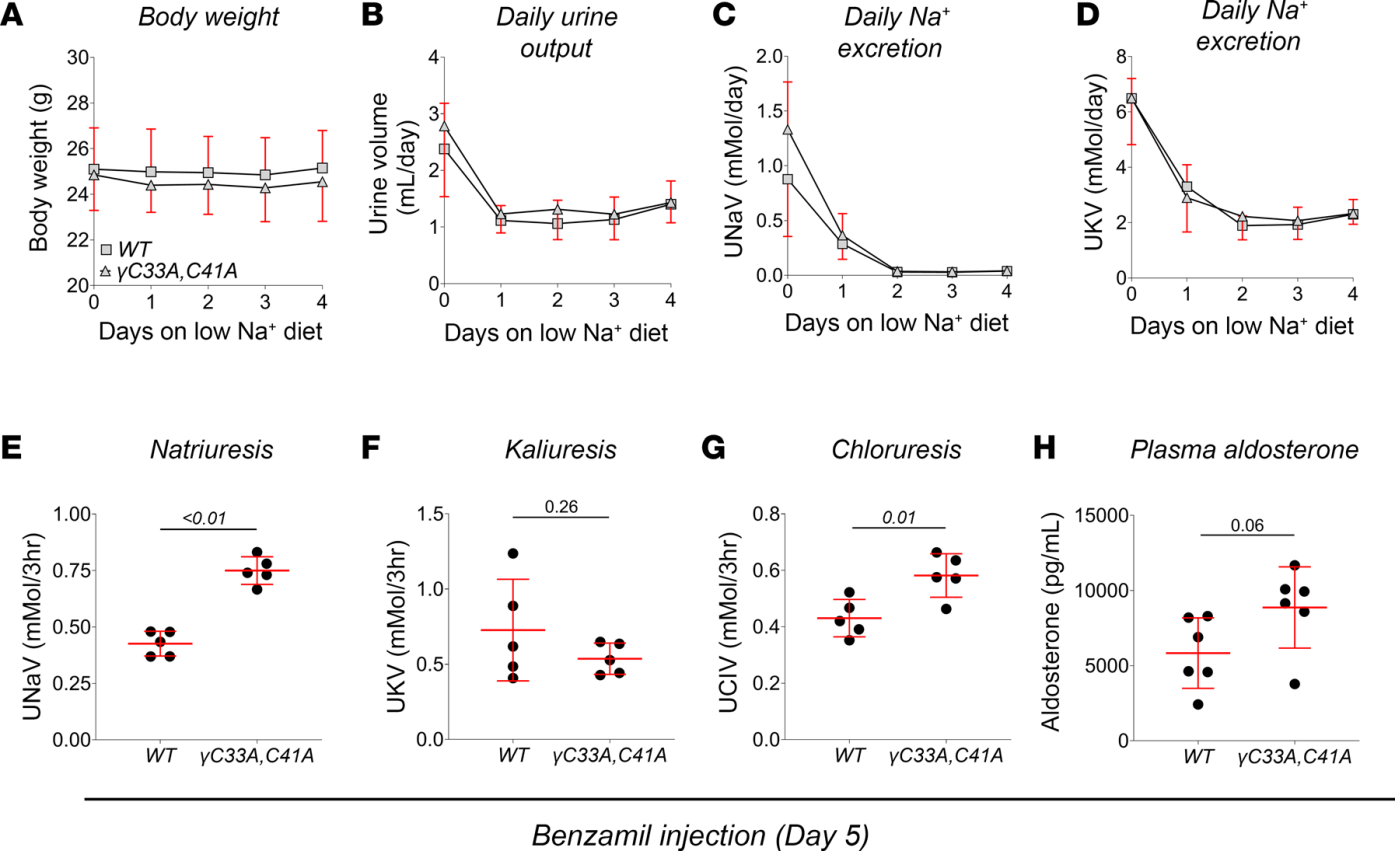
WT and γ^C33A,C41A^ mice exhibit robust benzamil-sensitive natriuresis following dietary Na^+^ restriction. (**A**–**D**) Body weight (**A**), daily urine output (**B**) and urinary Na^+^ (**C**) and K^+^ excretion (**D**) recorded from male WT (squares; *n* = 6) and γ^C33A,C41A^ mice (triangles; *n* = 6) over the course of 4 days on a low-Na^+^ diet. No statistical differences were detected for any parameter by 2-way ANOVA with repeated measures. (**E**–**G**) Urinary Na^+^ (**E**), K^+^ (**F**), and Cl^–^ (**G**) excretion from WT (*n* = 5) and γ^C33A,C41A^ (*n* = 5) mice in response to 1.5 mg/kg benzamil injection. Urines were collected over the first 3 hours following benzamil administration. (**H**) Plasma aldosterone levels measured in WT (*n* = 6) and γ^C33A,C41A^ (*n* = 6) mice following the benzamil injection experiment. Lines and error bars represent mean ± SD. Comparisons were made via 2-way ANOVA with repeated measures (**A**–**D**) or Student’s unpaired *t* test (**E**–**H**).

To evaluate the consequences of diminished ENaC activity on renal Na^+^ handling at the whole-organ level, we used metabolic cages to measure urinary volume and electrolyte composition before and during dietary Na^+^ restriction, as well as in response to selective ENaC blockade. Urinary Na^+^ excretion (UNaV) was similar between the groups at baseline and progressively diminished at similar rates during Na^+^ restriction (Figure 10C). Urinary K^+^ excretion (UKV) was also similar between genotypes throughout the experiment (Figure 10D). Likewise, body weight (Figure 10A) and effects on urine output over time (Figure 10B) were similar between groups. These data are consistent with the strong antidiuretic and antinatriuretic responses typical of dietary Na^+^ depletion.

In contrast with our patch clamp data, which clearly showed impaired ENaC function in γ^C33A,C41A^ mice, both groups responded to benzamil administration (1.5 mg/kg i.p.) with robust natriuresis (Figure 10E) and accompanying diuresis (0.6 ± 0.2 mL for both groups) over a 3-hour period. Even more surprising were the greater natriuretic (Figure 10E) and chloruretic (Figure 10G) responses to benzamil in γ^C33A,C41A^ versus WT mice. These data demonstrate that benzamil-sensitive Na^+^ transport was not only present, but also greater, in γ^C33A,C41A^ mice than in WT littermates under these conditions. Kaliuresis after benzamil was similar between groups (Figure 10F). Following the benzamil experiment, aldosterone levels in γ^C33A,C41A^ male mice may have been higher than WT (Figure 10H), although any difference did not reach significance (*P* = 0.06). However, aldosterone levels in both groups were substantially higher than previous experimental cohorts on a low-Na^+^ diet (Figure 4C and Figure 9B), which may reflect benzamil-induced diuresis and/or environmental stress induced by housing in the metabolic cages.

### WT and γ^C33A,C41A^ mice both display robust amiloride-sensitive Na^+^ absorption in the distal colon.

**Figure 11:**
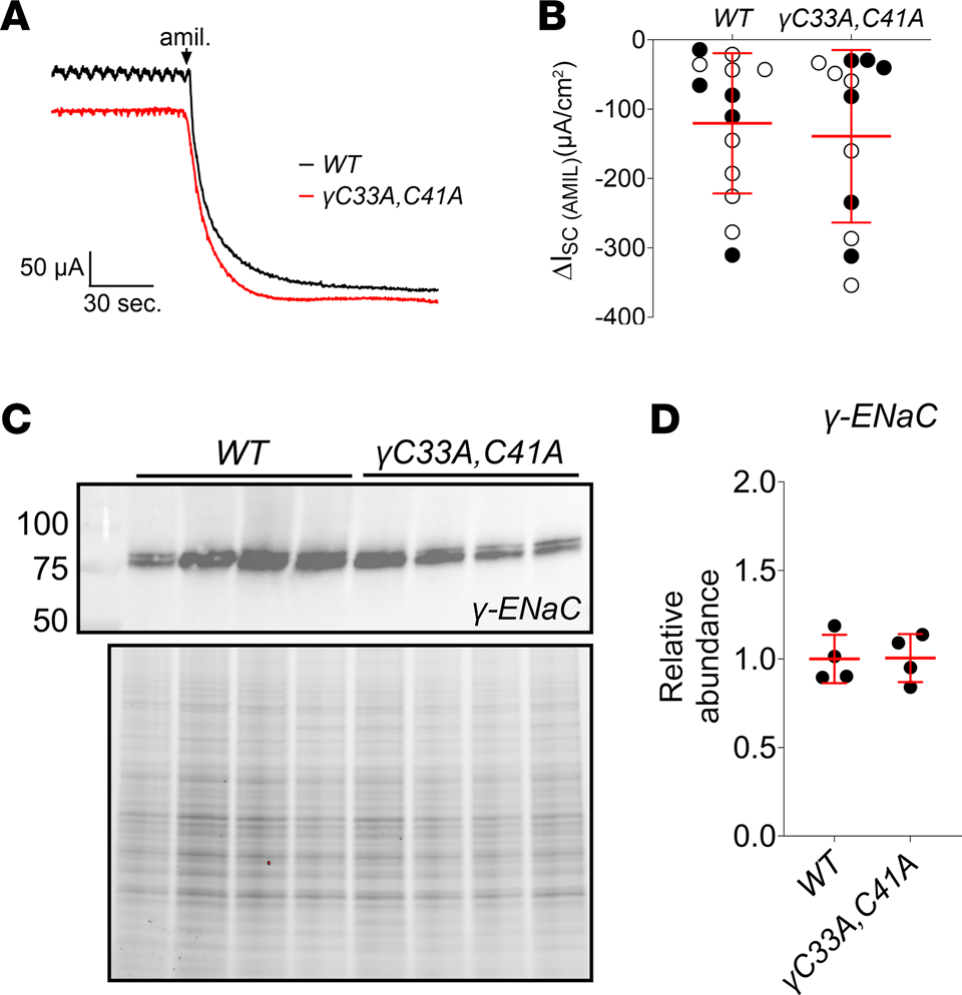
WT and γ^C33A,C41A^ mice exhibit amiloride-sensitive Na^+^ transport in the distal colon. (**A**) Representative short-circuit current (I_SC_) recordings of distal colons obtained from WT (black) and ENaCγ^C33A,C41A^ mice (red) maintained for 8 days on low-Na^+^ diet. Where indicated 100 μM amiloride was added to the apical chamber bath. (**B**) Amiloride-sensitive I_SC_ measured in WT (*n* = 13) and γ^C33A,C41A^ (*n* = 12) mouse distal colons. Data from male and female mice are shown as black and white circles, respectively. (**C**) Representative Western blot images showing the detection of the γENaC subunit in colon mucosal homogenates from male WT (*n* = 4) and γ^C33A,C41A^ (*n* = 4) mice maintained on a low-Na^+^ diet for 8 days. Standard molecular weight markers are shown in the far-left lane, along with corresponding weights, in kDa. Stain-free gel image showing total protein content for each sample is also provided. (**D**) Densitometric quantitation of γ subunit abundance was performed as described above. Lines and error bars represent mean ± SD. Statistical comparisons were made via Student’s unpaired *t* test and no significant differences were detected.

The distal colonic mucosa represents another major aldosterone-sensitive epithelium that participates in global electrolyte homeostasis (49–51). Here, aldosterone signaling induces the expression of ENaC β and γ subunits (α expression is constitutive), resulting in channel activity that can be readily measured as short-circuit current (I_SC_) in an Ussing chamber (52, 53). We found no differences between Na^+^-restricted WT and γ^C33A,C41A^ mice regarding their responses to another ENaC-selective blocker, amiloride (100 μM), when added to the mucosal bath (Figure 11, A and B). In distal colon, γ subunit expression was also similar between WT and γ^C33A,C41A^ mice under these conditions (Figure 11, C and D). Together, these data suggest that ENaC-dependent (amiloride/benzamil-sensitive) Na^+^ transport is readily observed in the kidneys and colons of γ^C33A,C41A^ mice, despite remarkably low ENaC activity in cell-attached recordings from isolated tubules.

### WT and γ^C33A,C41A^ mice express a benzamil-sensitive 20 pS channel in the CNT/CCD.

Our measurements of ENaC function at the single-channel level (split-open cell-attached recordings, Figures 2 and 5) and the whole-organ level (benzamil-induced natriuresis and amiloride-sensitive colonic I_SC_, Figures 10 and 11) are contradictory. Others have reported apical membrane, Na^+^-permeable channels in the distal nephron (54–56), as well as other ENaC-expressing epithelia (57–59), that display slow gating kinetics typical of the ENaC/Degenerin family. However, these channels are generally nonselective and usually have a unitary conductance of approximately 20 pS, much larger than that of typical ENaCs. In some cases, these nonselective channels were inhibited by amiloride with varying affinity (55).

**Figure 12:**
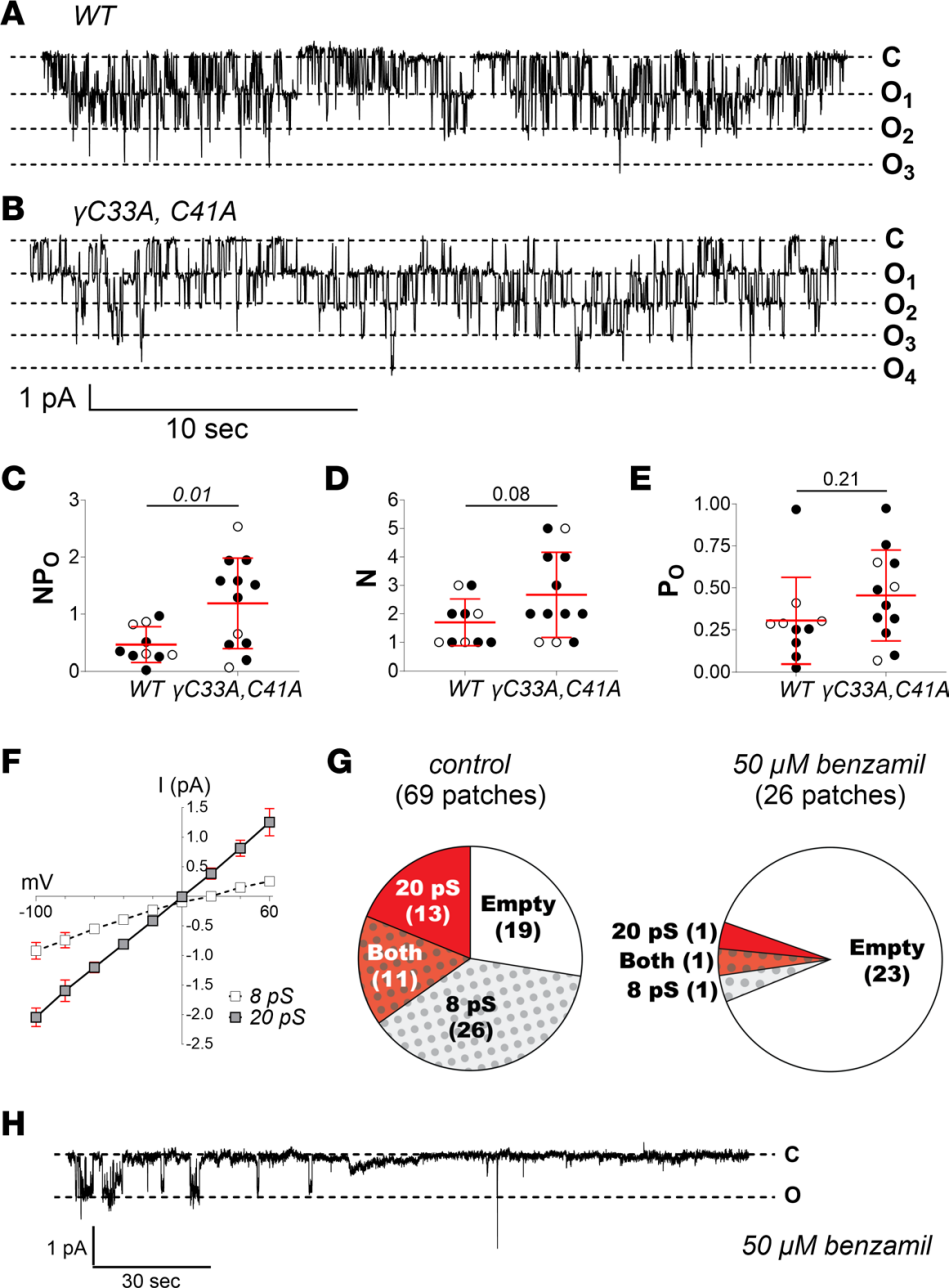
Presence of a benzamil-sensitive, 20 pS apical membrane conductance in CNT/CCDs of both WT and γ^C33A,C41A^ mice. (**A** and **B**) Representative recordings from cell-attached patches of PC apical membranes in split-open CNT/CCDs from WT (**A**) and γ^C33A,C41A^ mice (**B**) maintained on a low-Na^+^ diet for 8 days. Channel “closed” (C) and “open” (O) states are depicted to the right of each recording. (**C**–**E**) Total channel activity (*NP_O_*; **C**), number of observed channels per patch (*N*; **D**), and single-channel open probability (*P*_O_: **E**) in apical membrane patches from WT (*n* = 10) and γ^C33A,C41A^ (*n* = 12) mouse CNT/CCDs. Data from male and female mice are shown as black and white circles, respectively. (**F**) Current/voltage relationship of 8 pS and 20 pS channels observed in CNT/CCD apical membrane patches with Li^+^ in the patch pipette. (**G**) Frequency of channel appearances for both 8 and 20 pS conductance populations in the absence (left) or presence (right) of 50 μM benzamil in the bath and patch pipette solutions. (**H**) Representative recording of a 20 pS channel from a WT male mouse in the presence of benzamil, illustrating diminishing channel activity over time.

We also observed a 20 ± 0.3 pS (*n* = 12) channel in PC apical membrane patches from both WT and γ^C33A,C41A^ mice (Figure 12, A, B, and F). Interestingly, the activity (*NP_O_*) of this channel was greater in Na^+^-restricted γ^C33A,C41A^ compared with WT mice (Figure 12C). Given the presence of an inward Cl^–^ concentration gradient, current reversal at a holding potential of 0.1 ± 0.7 mV (*n* = 12) indicates that this is a nonselective cation channel. By comparison, the 8 pS channel displayed current reversal at 14 ± 2 mV holding potential (*n* = 13; *P* < 0.01 vs. 20 pS channel). Benzamil (50 μM) in the bath and patch pipette solutions decreased the likelihood of observing the 20 pS channel from 35% to 8% (*P* = 0.02; Mann-Whitney *U* test), as well as canonical 8 pS ENaC from 54% to 8% (*P* < 0.01; Mann-Whitney *U* test) (Figure 12G). Furthermore, when the 20 pS channel was observed in the presence of benzamil, its activity was low and diminished rapidly during the recording (example trace shown in Figure 12H), indicating that channel blockage increased with time. These data suggest that an apical membrane channel species, distinct from the canonical 8 pS conductance attributed to αβγ ENaC, may be involved in Na^+^ handling within the ASDN. This may also explain our finding of benzamil-sensitive natriuresis in γ^C33A,C41A^ mice despite markedly reduced *P_O_* of prototypic ENaC.

## Discussion

Previous studies established that palmitoylation of the γ subunit at C33 and C41 activates ENaC in vitro. Disrupting palmitoylation at these sites markedly reduces channel activity and prevents palmitoyltransferase-dependent channel activation when expressed in a heterologous system (37). To date, the broader physiological importance of this regulatory mechanism has been unclear. We now demonstrate that mice lacking these N-terminal cysteines (γ^C33A,C41A^ mice) express ENaCs in the ASDN with greatly reduced activity compared with WT littermates.

Cell-attached recordings from split-open CNT/CCDs revealed that single-channel *P_O_*, as well as *NP_O_*, were significantly reduced in γ^C33A,C41A^ compared with WT mice receiving a control diet (Figure 2). This finding is consistent with data from 2-electrode voltage clamp and patch clamp experiments using *Xenopus* oocytes expressing channels that carry the same mutations (37). We further observed that channel activity in γ^C33A,C41A^ mice remained quite low under dietary Na^+^-restricted conditions (Figure 5), suggesting that a loss of function was not overcome by the effects of aldosterone.

Na^+^ restriction induced higher aldosterone levels in male γ^C33A,C41A^ versus WT mice (Figure 4), which was likely a compensatory response to impaired ENaC function, although we did not observe a similar effect in females. Sex differences associated with epithelial ion transport regulation are known to play a major role in the adaptive responses to various dietary manipulations (40, 60–63). Increases in aldosterone levels in response to Na^+^ restriction are more modest in female versus male rats (64). We found that NCC and NKCC2 expression were approximately 50% greater in female versus male mice of either genotype (Figure 8), suggesting an enhanced capacity for Na^+^ reabsorption upstream of the CCD. Thus, females may require less aldosterone to maintain homeostasis, which would be consistent with the observed sex-dependent effect of Na^+^ restriction on aldosterone production between γ^C33A,C41A^ and WT mice.

Patch clamp experiments showed that prototypic ENaC function was clearly diminished in γ^C33A,C41A^ mice, despite no apparent changes in channel subunit expression or proteolysis (Figure 7). Because γ subunit cleavage at the distal site is thought to occur after channels traffic to the apical membrane (65), our biochemical findings further suggest that ENaC surface expression was similar in γ^C33A,C41A^ and WT mice. These data accord with results from WT and γ^C33A,C41A^ channels expressed in *Xenopus* oocytes (37). However, a direct assessment of ENaC surface expression (e.g., in vivo biotinylation of surface proteins in mouse tubules) was not performed. We therefore rest on the assumption that the abundance of double-cleaved γENaC approximates that of the surface-localized channel pool.

Surprisingly, when we examined Na^+^ transport at the whole-organ level, we found that WT and γ^C33A,C41A^ mice both exhibited robust benzamil-induced natriuresis (Figure 10), as well as amiloride-sensitive I_SC_ in the distal colon (Figure 11). Thus, the response to classical ENaC-selective blockers remains intact in γ^C33A,C41A^ mice, despite a profound reduction in *P_O_* and no apparent increase in channel expression. Further, K^+^ handling in response to acute or chronic K^+^ loading was not impaired in γ^C33A,C41A^ mice (Figure 9), indicating these animals possess a sufficient capacity for electrogenic Na^+^/K^+^ exchange in the ASDN. How can these conflicting observations be reconciled?

One possible explanation is that other benzamil/amiloride-sensitive transport mechanisms, aside from prototypic ENaCs, contribute to Na^+^ reabsorption in the ASDN. We observed a second cation channel species in PC apical membranes that displays unitary conductance larger than that of ENaC (20 vs. 8 pS with Li^+^ as the charge carrier) but was blocked by 50 μM benzamil (Figure 12). This 20 pS channel is likely nonselective, given its reversal potential of near 0 mV in cell-attached patches. However, opening of a nonselective cation channel at the PC apical membrane would still heavily favor Na^+^ entry from the tubule lumen as a result of the attendant electrochemical driving forces. Blockage of such a channel could significantly impact Na^+^ transport in the ASDN, especially considering the relatively high *P_O_* and unitary conductance, as well as the frequency at which these channels were observed.

Interestingly, the activity of this 20 pS channel was greater in Na^+^-restricted γ^C33A,C41A^ mice compared with WT littermates, raising the possibility that it may have a compensatory role when prototypic ENaC activity is compromised. We cannot determine the molecular identity of the 20 pS nonselective cation channel observed in our experiments based on the current data. The role of this channel in tubular Na^+^ reabsorption therefore remains to be defined. However, future studies will be aimed at exploring the composition and physiological importance of this channel in greater detail.

Other ENaC-like, amiloride-sensitive cation channels with diverse biophysical characteristics have been described in transporting epithelia (54–56, 58). For example, alveolar epithelial cells express a nonselective cation channel species that, when compared with typical ENaCs, exhibits shorter open/closed times, is approximately 100 times less sensitive to amiloride, and has a larger unitary conductance of around 20 pS with Na^+^ as the charge carrier (57). Eaton and colleagues provide evidence that these nonselective cation channels comprise ENaCα and acid-sensing ion channel (ASIC1a) subunits. ENaCα/ASIC1a channels were further shown to have an important role in alveolar fluid clearance, which depends on vectorial ion transport.

In the kidney, Fila et. al. recently demonstrated that a novel splice variant of ASIC2 is amiloride sensitive and contributes to Na^+^ retention in a rodent model of nephrotic syndrome (66), a phenomenon that was previously considered to be largely mediated by ENaC (67–69). Other ~20 pS cation channels have also been documented in patch clamp recordings of native collecting duct PCs (70) and cultured collecting duct cell lines (71–73). In mouse CCDs, such a channel was observed in PC apical membrane patches from animals lacking primary cilia (70). Activity of this channel was increased in ciliopathic mice compared with WT littermates. This was concurrent with increased abundance of TRPV4 and TRPP3 mRNA, although the authors did not demonstrate a functional connection. Interestingly, TRPP3 is sensitive to amiloride and benzamil (74). TRPM4, another nonselective, 23 pS cation channel, was also identified in mpkCCD cell apical membrane patches (75). A functional role for the aforementioned TRP channels with respect to Na^+^ transport in the CCD has not yet been determined.

Our experimental model system is a constitutive disruption of known palmitoylation sites, rather than an inducible mechanism. Therefore, γ^C33A,C41A^ mice carry these mutations throughout development and postnatal maturation. Nephron development may be affected in these mutants to compensate for diminished ENaC function in the distal tubule. However, our analysis of transporter protein expression in kidney lysates indicates that distal convoluted tubule– or thick ascending limb–localized Na^+^ transport mechanisms are not upregulated in γ^C33A,C41A^ mice (Figure 8). If upstream tubule segment remodeling occurs in γ^C33A,C41A^ mice (e.g., hyperplasia or hypertrophy), such adaptations were not evident in these studies.

In agreement with in vitro studies, we observed that prototypic ENaCs in the CNT/CCDs of γ^C33A,C41A^ exhibited a dramatically reduced *P_O_* compared with WT littermates. While our results suggest that ENaC palmitoylation has an important role in activating the channel in vivo, we cannot provide direct evidence that the γ subunit is modified by palmitoylation in the kidney. We previously used fatty acid exchange chemistry to demonstrate palmitoylation of N- and C-terminally tagged β and γ subunits using heterologous expression systems (36, 37). To date, we have not been successful in using a similar approach to demonstrate ENaC palmitoylation in native (untagged) proteins.

A recently developed technique to detect palmitoylated cysteine residues involves replacing the acyl group with a large polyethylene glycol, resulting in an upward shift in the target protein when assayed by immunoblot (76). Our attempts at using this approach were also unsuccessful, likely because the palmitoylated cysteines in γENaC reside within the N-terminal segment, which is cleaved during channel processing. Due to the location of the epitope sequence targeted by the γENaC antibody used here (residues 629–650 in the C-terminus), the mature N-terminal fragment that contains the palmitoylated cysteines cannot be visualized by immunoblotting. We therefore cannot rule out the possibility that loss of γ subunit N-terminal cysteines confers a reduction in ENaC activity independent of palmitoylation. For example, Kellenberger et. al. suggested that intracellular cysteines regulate ENaC function through redox chemistry (77). Reducing or oxidizing reagents enhanced or inhibited ENaC activity in excised membrane patches, respectively. Collective mutation of all intracellular cysteines in the α, β, and γ subunits attenuated these responses. We cannot exclude the possibility that a similar mechanism is at least partially responsible for the loss of function observed in γ^C33A,C41A^ mice.

Evaluating the consequences of a total loss of ENaC function is made difficult by the fact that constitutive knockout of ENaC subunits causes perinatal lethality due to impaired fluid clearance in the lung and severe salt wasting (78–80). Hummler and colleagues developed a series of inducible, nephron-specific ENaC-knockout mouse models as a strategy to circumvent this issue (81–84). Mice with induced γ subunit knockout exhibit a PHA-1–like phenotype with salt wasting, weight loss, hyperkalemia, and elevated aldosterone. This may reflect a downregulation on NCC as well as ENaC, given that urinary Na^+^ wasting and weight loss were not observed in mice maintained on a K^+^-deficient (<0.1% K^+^) diet (82, 85). Here, we show that γ^C33A,C41A^ mice display a profound loss of prototypic ENaC activity in the ASDN but have no overt phenotype. It is therefore unlikely that the γ^C33A,C41A^ mutation described here is functionally similar to a knockout of the γ subunit. Single-channel recordings from inducible γ subunit–knockout mice were not reported but may yield important insights into Na^+^ transport in the ASDN, including the possible involvement of other channels.

In conclusion, our study demonstrates that 2 key cysteine residues in the γ subunit N-terminus, γC33 and γC41, are required for robust ENaC activity in vivo. However, a benzamil-sensitive, nonselective cation channel in the ASDN may compensate for the loss of prototypic ENaC function caused by mutation of these residues (γ^C33A,C41A^).

## Methods

### Animals.

γ^C33A,C41A^ mice were generated on a 129SVE background using CRISPR/Cas9 technology, with the assistance of the mouse embryonic services core facility at the University of Pittsburgh Department of Immunology. Alanine substitutions at cysteines 33 and 41 (γ^C33A,C41A^) were induced via homology-directed repair (HDR). Embryos were injected with single-guide RNA (50 ng/μL; 5′-TAATACGACTCACTATAGGCACGATGCGCCGGCAGCCGTGTTTTAGAGCTAGAAATAGCA-3′), Cas9 mRNA (100 ng/μL), and single-stranded oligo DNA nucleotides for HDR (50 μM; 5′-AAGAATCTGCCAGTTCGAGGCCCCCAGGCACCGACCATTAAGGACCTGATGCATTGGTACGCGTTGAACACCAACACCCACGGAGCTCGGCGCATCGTGGTGTCCCGAGGCCGCCTTCGGCGCCTGTTGTGGATTGCGTTCACGCTG-3′). *Mlu1* restriction sites were introduced into γ^C33A,C41A^ mice near A33 such that digestion of PCR-amplified products yielded fragments of 395 bp in WT and 195/205 bp in mutant mice, respectively (Figure 1). Founder mice were genotyped by PCR (forward primer 5′-GACATAGGGCTGACACACCA-3′, reverse primer 5′-CCCACCAGTTTCTTCGACTCA-3′) and confirmed by sequencing the amplification products. These mice were backcrossed against WT 129SVE mice for 3 generations before arranging heterozygous mating schemes for producing experimental cohorts. After establishing colonies, mice were moved to Charles River Laboratories for breeding and genotyping. Experimental cohorts were then shipped to the University of Pittsburgh for experiments and genotype confirmation.

### Blood electrolyte and aldosterone measurements.

Mice were anesthetized with isoflurane and blood was collected via left ventricular puncture or via retro-orbital sinus bleed. Whole blood was analyzed with an iStat analyzer (Abbott Laboratories) using chem8+ cartridges to gather electrolyte and metabolic parameters. For left ventricular punctures, remaining blood was centrifuged for 15 minutes at 1,500*g* at 4°C to isolate the plasma. Plasma aldosterone was measured via ELISA (IBL-America IB79134) according to the manufacturer’s instructions.

### Immunoblotting.

Kidney homogenates were prepared by lysing tissues in CelLytic MT lysis buffer (MilliporeSigma; 5% weight/volume) using a glass homogenizer. After determining protein concentrations for each lysate, 30 mg of total protein was resolved using 4%–15% TGX-Stain–free polyacrylamide gels (BioRad). For some experiments, N-linked glycosylation products were removed using PNGase F (New England Biolabs P0704) prior to gel loading, as per the manufacturer’s instructions. Uniform protein loading between samples was confirmed after electrophoresis by imaging the stain-free gels using a Chemidoc imager (BioRad), before transferring the resolved proteins to PVDF membranes (BioRad). After transfer, membranes were incubated in blocking buffer (5% milk dissolved in Tris-buffered saline with 0.1% Tween 20; TBST) for 2 hours at room temperature. Membranes were then incubated at 4°C overnight in blocking buffer containing primary antibodies against the ENaC α subunit (gift from Johannes Loffing, University of Zurich, Zurich, Switzerland; 1:1,000; raised against mouse αENaC residues 2–21) (86), ENaC γ subunit (Stressmarq SPC-405; 1:1,000; raised against rat γENaC residues 629–650), NCC (David Ellison, Oregon Health & Science University, Portland, Oregon, USA; 1:5,000; raised against mouse NCC residues 1–98) (87), phospho-NCC (Phosphosolutions p1311-53, 1:5,000; raised against residues surrounding pThr53 in mouse NCC), or NKCC2 (Stressmarq SPC-401; 1,1000; raised against rat NKCC2 residues 33–55). The following day, membranes were washed 4 times, 10 minutes each, with TBST and incubated in blocking buffer containing HRP-conjugated secondary antibody (Thermo Fisher Scientific 31460; goat anti-rabbit, 1:10,000) for 1 hour at room temperature. Target proteins were detected by chemiluminescence using Clarity peroxide substrate reagent (BioRad). Blots were imaged with a Chemidoc imager, and protein abundance was quantified by measuring the signal intensity via ImageJ software (NIH) and normalizing to the total protein signal detected in each sample from corresponding stain-free gel images.

### Patch clamp electrophysiology.

Freshly isolated kidneys were sliced into approximately 1 mm sections and rinsed thoroughly in warm L-15 medium (Gibco). Using a stereomicroscope, small portions of the renal cortex were separated from the medulla and incubated in type II collagenase (MilliporeSigma C2-22; 1 mg/mL) for 40 minutes at 37°C. After incubation, tissue segments were rinsed 5 consecutive times in ice-cold L-15 medium to stop enzymatic digestion. CNT/CCDs were isolated using very fine needles under a stereomicroscope. CNT/CCD segments were identified based on the following spatial/morphological features: 1) being located just downstream of distal convolutions, 2) coarse appearance, 3) presence of branched segments, and 4) medium-sized tubule diameter (i.e., between that of the proximal tubule and loop of Henle segments). DCT2/CNTs were identified as the last third of the convoluted segment and the tubule areas upstream of branching points.

Tubules were mounted to poly-l-lysine–coated coverslips and transferred to a perfusion/recording chamber continuously perfused with physiological saline solution (in mM: 140 Na^+^, 4.5 K^+^, 2 Ca^2+^, 1.2 Mg^2+^, 150.9 Cl^–^, 5 glucose, 10 HEPES, 5 Tris, pH 7.4) at a rate of ~1 mL/min. Immobilized tubules were split open using sharpened micropipettes controlled by a micromanipulator (Sutter Instruments), to gain access to the cells’ apical membranes. PCs were identified based on the appearance of a small, flat apical membrane surface and a prominent nucleus. Apical membrane seals were made using micropipettes pulled from borosilicate glass (Sutter Instruments), polished to give a series resistance of 5–8 MΩ, and filled with LiCl solution (in mM: 140 Li^+^, 144 Cl^–^, 2 Mg^+^, 10 HEPES, pH 7.4). High-resistance seals (>2 GΩ) were made by applying gentle suction to the pipette using a glass syringe. Membrane currents were recorded in a cell-attached configuration using a PC-One Patch Clamp amplifier (Dagan Corp.), operated by pClamp 10 software (Molecular Devices). Currents were recorded at a sampling frequency of 5 kHz and filtered at 100 Hz by a built-in 4-pole low-pass Bessel filter. All recordings were manually inspected and corrected for any baseline drift prior to analyzing channel transitions via single-channel event detection in Clampfit software. Channel transition events were then measured over a period of at least 2 minutes. All analyses were again visually inspected prior to inclusion of the data.

### Body composition analysis.

Total body water content in restrained mice was determined using a quantitative magnetic resonance imager (EchoMRI-100H). Measurements were performed in duplicate for each mouse during the course of dietary Na^+^ restriction. Total body water mass (including free water) was taken as a percentage of total body weight to yield percentage body water values. All measurements were taken in 24-hour intervals.

### K^+^ gavage experiments.

Mice maintained on standard chow were administered 150 μL of a solution containing 5% KCl and 2% sucrose via gastric gavage. At 30- and 60-minute time points, mice were briefly anesthetized with 5% isoflurane, and blood samples were collected via retro-orbital bleed. Blood K^+^ values were measured using an iStat as described above.

### Ussing chamber electrophysiology.

Colons were harvested from anesthetized mice, and mucosa/submucosa preparations were made by blunt dissection. The colon was opened longitudinally along the mesenteric line and pinned down in a sylgard dish with the mucosal surface facing downward. Using a razor blade, a superficial lateral incision was made near the rectum, allowing separation of the mucosa/submucosa and muscularis propria. The tissue layers were then teased apart using fine forceps under a dissecting microscope. Tissues were mounted on custom-made sliders (aperture 0.3 cm^2^) for use in an Ussing-style recording chamber (Physiologic Instruments) and were bathed in Ringer’s solution containing the following, in mM: 140 Na^+^, 119.8 Cl^–^, 25 HCO_3_^–^, 5.2 K^+^, 1.2 Ca^2+^, 1.2 Mg^2+^, 2.4 HPO_4_^2–^, 0.4 H_2_PO_4_^–^, and 10 glucose. The solution was continuously bubbled with 5% CO_2_ balanced with oxygen to maintain pH at 7.41 and gradually warmed to 37°C using a circulating heated water bath. Tetrodotoxin (0.5 μM) was added to the serosal bath to suppress residual neurogenic activity. Trans-epithelial voltage (V_TE_) was monitored using a multichannel voltage clamp/amplifier (VCC MC6; Physiologic Instruments) until the tissues stabilized. V_TE_ was then clamped to 0 mV using computer-operated software (pClamp 10, Molecular Devices) to measure I_SC_. After currents stabilized, ENaC activity was measured as the change in I_SC_ after addition of 100 μM amiloride to the apical bath.

### Metabolic cage experiments.

For baseline urine collections, mice were singly housed in metabolic cages (Techniplast) for an acclimation period of 3 days with free access to food and water. The standard pelleted diet was replaced with a gel mixture (pelleted diet blended with 3% agar) to prevent contamination of the urine by pellet debris. After acclimation, baseline urines were collected over 24 hours, and Na^+^ and K^+^ concentrations were measured with an EasyLyte analyzer (Medica). Total urine output (in milliliters) was also recorded for the 24-hour period, which was used to calculate rates of UNaV and UKV. For benzamil response experiments, mice were maintained on a low-Na^+^ gel diet for 5 days to enhance ENaC expression. This diet was prepared in the same manner as normal gel diet but with <0.01% Na^+^ chow (Teklad) instead of standard chow. Body weight, daily urine output, and UNaV/UKV were monitored over the treatment period. After 5 days of Na^+^ restriction, each mouse was injected i.p. with 1.5 mg/kg benzamil (dissolved in sterile saline + 0.1% DMSO), and urine was collected over the following 3 hours. Following the experiment, mice were euthanized, and blood was collected for aldosterone measurements.

### Statistics.

Methods of statistical analysis are described in detail in the text and/or figure legends. Endpoint comparisons between genotypes were made via unpaired 2-tailed Student’s *t* test. Time course comparisons between genotypes were made via 2-way ANOVA with repeated measures. Sex- and genotype-dependent comparisons were made via 2-way ANOVA with Tukey’s post hoc. Comparison of observed channel appearances between treatment groups was performed via Mann-Whitney *U* test. For all analyses, *P* < 0.05 was considered significant.

### Study approval.

Animal protocols were approved by the University of Pittsburgh Institutional Animal Care and Use Committee.

### Data availability.

All data presented in this manuscript are accessible in the Supporting Data Values (supplemental material available online with this article; https://doi.org/10.1172/jci.insight.172051DS1) XLS file or by request to the corresponding author.

## Author contributions

AJN designed and performed experiments, analyzed data, and drafted and revised the manuscript. SMM performed experiments. SS assisted with mouse model generation, performed experiments, and edited the manuscript. NAC performed experiments and analyzed data. ECR assisted with mouse model generation and data analysis. OBK assisted with data analysis and edited the manuscript. MDC assisted with data analysis and edited the manuscript. ALM assisted with experiments and mouse breeding. AW assisted with mouse breeding. SG assisted with mouse model generation. AK assisted with data analysis and edited the manuscript. RPH assisted with data analysis and edited the manuscript. TRK conceived of the study, designed experiments, analyzed data, and revised the manuscript.

## Supplementary Material

Supplemental data

Supporting data values

## Figures and Tables

**Table 1:**
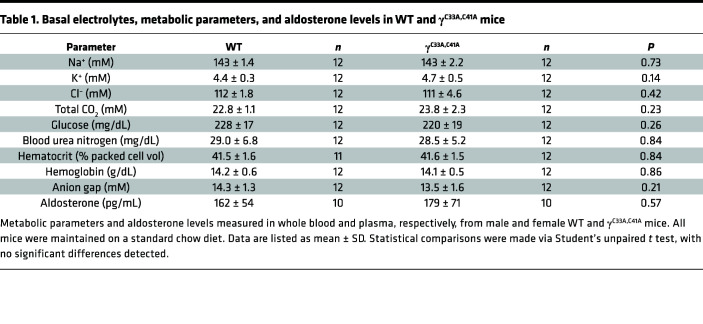
Basal electrolytes, metabolic parameters, and aldosterone levels in WT and γ^C33A,C41A^ mice

**Table 2:**
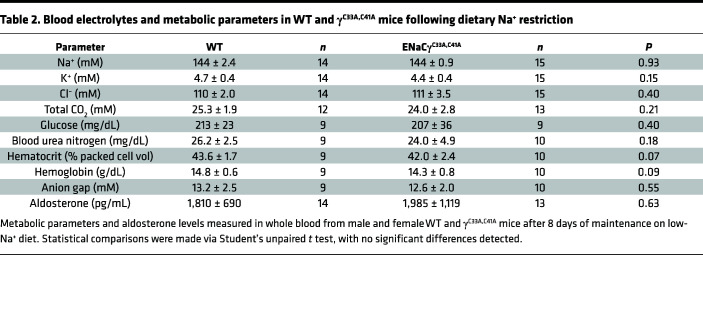
Blood electrolytes and metabolic parameters in WT and γ^C33A,C41A^ mice following dietary Na^+^ restriction

## Version 1 09/14/2023

In-Press Preview

## Version 2 11/08/2023

Electronic publication

## References

[1] Rotin D, Staub O (2021). Function and regulation of the epithelial Na^+^ channel ENaC. Compr Physiol.

[2] Palmer LG, Schnermann J (2015). Integrated control of Na transport along the nephron. Clin J Am Soc Nephrol.

[3] Mutchler SM (2021). Epithelial sodium channel and salt-sensitive hypertension. Hypertension.

[4] Rossier BC (2014). Epithelial sodium channel (ENaC) and the control of blood pressure. Curr Opin Pharmacol.

[5] Palmer LG, Frindt G (1986). Amiloride-sensitive Na channels from the apical membrane of the rat cortical collecting tubule. Proc Natl Acad Sci U S A.

[6] Bhalla V, Hallows KR (2008). Mechanisms of ENaC regulation and clinical implications. J Am Soc Nephrol.

[7] Pearce D (2015). Collecting duct principal cell transport processes and their regulation. Clin J Am Soc Nephrol.

[8] Nesterov V (2012). Aldosterone-dependent and -independent regulation of the epithelial sodium channel (ENaC) in mouse distal nephron. Am J Physiol Renal Physiol.

[9] Bugaj V (2009). Activation of the epithelial Na+ channel in the collecting duct by vasopressin contributes to water reabsorption. Am J Physiol Renal Physiol.

[10] Sun P (2012). Angiotensin II stimulates epithelial sodium channels in the cortical collecting duct of the rat kidney. Am J Physiol Renal Physiol.

[11] Frindt G (2009). K+ secretion in the rat kidney: Na+ channel-dependent and -independent mechanisms. Am J Physiol Renal Physiol.

[12] Kucher V (2011). Voltage-dependent gating underlies loss of ENaC function in pseudohypoaldosteronism type 1. Biophys J.

[13] Schild L (1996). The ENaC channel as the primary determinant of two human diseases: Liddle syndrome and pseudohypoaldosteronism. Nephrologie.

[14] Tajima T (2017). Clinical features and molecular basis of pseudohypoaldosteronism type 1. Clin Pediatr Endocrinol.

[15] Enslow BT (2019). Liddle’s syndrome mechanisms, diagnosis and management. Integr Blood Press Control.

[16] Schild L (1996). Identification of a PY motif in the epithelial Na channel subunits as a target sequence for mutations causing channel activation found in Liddle syndrome. EMBO J.

[17] GenSalt Collaborative Research Group (2007). GenSalt: rationale, design, methods and baseline characteristics of study participants. J Hum Hypertens.

[18] Ray EC (2016). Human epithelial Na+ channel missense variants identified in the GenSalt study alter channel activity. Am J Physiol Renal Physiol.

[19] Chen J (2013). Gain-of-function variant of the human epithelial sodium channel. Am J Physiol Renal Physiol.

[20] Gu X (2018). Resequencing epithelial sodium channel genes identifies rare variants associated with blood pressure salt-sensitivity: the GenSalt study. Am J Hypertens.

[21] Pitzer AL (2020). ENaC in salt-sensitive hypertension: kidney and beyond. Curr Hypertens Rep.

[22] Blobner BM (2022). Rare variants in genes encoding subunits of the epithelial Na^+^ channel are associated with blood pressure and kidney function. Hypertension.

[23] Kleyman TR, Eaton DC (2020). Regulating ENaC’s gate. Am J Physiol Cell Physiol.

[24] Flores SY (2005). Aldosterone-induced serum and glucocorticoid-induced kinase 1 expression is accompanied by Nedd4-2 phosphorylation and increased Na+ transport in cortical collecting duct cells. J Am Soc Nephrol.

[25] Loffing J (2001). Aldosterone induces rapid apical translocation of ENaC in early portion of renal collecting system: possible role of SGK. Am J Physiol Renal Physiol.

[26] Rossier BC, Stutts MJ (2009). Activation of the epithelial sodium channel (ENaC) by serine proteases. Annu Rev Physiol.

[27] Sheng S (2006). Furin cleavage activates the epithelial Na+ channel by relieving Na+ self-inhibition. Am J Physiol Renal Physiol.

[28] Bruns JB (2007). Epithelial Na+ channels are fully activated by furin- and prostasin-dependent release of an inhibitory peptide from the gamma-subunit. J Biol Chem.

[29] Archer CR (2020). Phosphatidylinositol 4,5-bisphosphate directly interacts with the β and γ subunits of the sodium channel ENaC. J Biol Chem.

[30] Pochynyuk O (2007). Binding and direct activation of the epithelial Na+ channel (ENaC) by phosphatidylinositides. J Physiol.

[31] Helms MN (2005). Phosphatidylinositol 3,4,5-trisphosphate mediates aldosterone stimulation of epithelial sodium channel (ENaC) and interacts with gamma-ENaC. J Biol Chem.

[32] Bize V, Horisberger JD (2007). Sodium self-inhibition of human epithelial sodium channel: selectivity and affinity of the extracellular sodium sensing site. Am J Physiol Renal Physiol.

[33] Kashlan OB (2015). Na+ inhibits the epithelial Na+ channel by binding to a site in an extracellular acidic cleft. J Biol Chem.

[34] Collier DM, Snyder PM (2009). Extracellular protons regulate human ENaC by modulating Na+ self-inhibition. J Biol Chem.

[35] Carattino MD (2004). Epithelial Na+ channels are activated by laminar shear stress. J Biol Chem.

[36] Mueller GM (2010). Cys palmitoylation of the beta subunit modulates gating of the epithelial sodium channel. J Biol Chem.

[37] Mukherjee A (2014). Cysteine palmitoylation of the γ subunit has a dominant role in modulating activity of the epithelial sodium channel. J Biol Chem.

[38] Mukherjee A (2017). Specific palmitoyltransferases associate with and activate the epithelial sodium channel. J Biol Chem.

[39] Asher C (1996). Aldosterone-induced increase in the abundance of Na+ channel subunits. Am J Physiol.

[40] Hu R (2020). Sex differences in solute transport along the nephrons: effects of Na^+^ transport inhibition. Am J Physiol Renal Physiol.

[41] Nesterov V (2021). Critical role of the mineralocorticoid receptor in aldosterone-dependent and aldosterone-independent regulation of ENaC in the distal nephron. Am J Physiol Renal Physiol.

[42] Uchimura K (2012). In vivo contribution of serine proteases to the proteolytic activation of γENaC in aldosterone-infused rats. Am J Physiol Renal Physiol.

[43] Frindt G (2018). Na restriction activates epithelial Na channels in rat kidney through two mechanisms and decreases distal Na^+^ delivery. J Physiol.

[44] Zachar RM (2015). The epithelial sodium channel γ-subunit is processed proteolytically in human kidney. J Am Soc Nephrol.

[45] Carattino MD (2008). Proteolytic processing of the epithelial sodium channel gamma subunit has a dominant role in channel activation. J Biol Chem.

[46] Balchak DM (2018). The epithelial Na^+^ channel gamma subunit autoinhibitory tract suppresses channel activity by binding the gamma subunit’s finger-thumb domain interface. J Biol Chem.

[47] Frindt G (2021). Cleavage state of γENaC in mouse and rat kidneys. Am J Physiol Renal Physiol.

[48] Maddox RW (1985). Extreme hyperkalemia associated with amiloride. South Med J.

[49] Bertog M (2008). Aldosterone responsiveness of the epithelial sodium channel (ENaC) in colon is increased in a mouse model for Liddle’s syndrome. J Physiol.

[50] Epple HJ (2000). Early aldosterone effect in distal colon by transcriptional regulation of ENaC subunits. Am J Physiol Gastrointest Liver Physiol.

[51] Harvey BJ (2008). Rapid responses to aldosterone in the kidney and colon. J Steroid Biochem Mol Biol.

[52] Greig ER (2002). Segmental variability of ENaC subunit expression in rat colon during dietary sodium depletion. Pflugers Arch.

[53] Malsure S (2014). Colon-specific deletion of epithelial sodium channel causes sodium loss and aldosterone resistance. J Am Soc Nephrol.

[54] Eaton DC (1995). Renal sodium channels: regulation and single channel properties. Kidney Int.

[55] Benos DJ (1996). Diversity and regulation of amiloride-sensitive Na+ channels. Kidney Int.

[56] Nonaka T (1995). Monovalent cation selective channel in the apical membrane of rat inner medullary collecting duct cells in primary culture. Biochim Biophys Acta.

[57] Trac PT (2017). Alveolar nonselective channels are ASIC1a/α-ENaC channels and contribute to AFC. Am J Physiol Lung Cell Mol Physiol.

[58] Lazrak A (2023). Low molecular weight hyaluronan inhibits lung epithelial ion channels by activating the calcium-sensing receptor. Matrix Biol.

[59] Gogelein H, Capek K (1990). Quinine inhibits chloride and nonselective cation channels in isolated rat distal colon cells. Biochim Biophys Acta.

[60] Soliman RH (2022). Sex differences in diurnal sodium handling during diet-induced obesity in rats. Hypertension.

[61] Veiras LC (2017). Sexual dimorphic pattern of renal transporters and electrolyte homeostasis. J Am Soc Nephrol.

[62] Faulkner JL (2018). Lack of suppression of aldosterone production leads to salt-sensitive hypertension in female but not male Balb/C mice. Hypertension.

[63] Zhang D (2020). Loss of circadian gene Bmal1 in the collecting duct lowers blood pressure in male, but not female, mice. Am J Physiol Renal Physiol.

[64] Nickerson AJ, Rajendran VM (2021). Aldosterone up-regulates basolateral Na^+^ -K^+^ -2Cl^-^ cotransporter-1 to support enhanced large-conductance K^+^ channel-mediated K^+^ secretion in rat distal colon. FASEB J.

[65] Frindt G, Palmer LG (2009). Surface expression of sodium channels and transporters in rat kidney: effects of dietary sodium. Am J Physiol Renal Physiol.

[66] Fila M (2021). A variant of ASIC2 mediates sodium retention in nephrotic syndrome. JCI Insight.

[67] Bohnert BN (2018). Aprotinin prevents proteolytic epithelial sodium channel (ENaC) activation and volume retention in nephrotic syndrome. Kidney Int.

[68] Pearce D (2022). Regulation of distal tubule sodium transport: mechanisms and roles in homeostasis and pathophysiology. Pflugers Arch.

[69] Hinrichs GR (2020). Mechanisms of sodium retention in nephrotic syndrome. Curr Opin Nephrol Hypertens.

[70] Saigusa T (2019). Loss of primary cilia increases polycystin-2 and TRPV4 and the appearance of a nonselective cation channel in the mouse cortical collecting duct. Am J Physiol Renal Physiol.

[71] Isaeva E (2022). Crosstalk between epithelial sodium channels (ENaC) and basolateral potassium channels (K_ir_ 4.1/K_ir_ 5.1) in the cortical collecting duct. Br J Pharmacol.

[72] Ilatovskaya DV (2011). Cortical actin binding protein cortactin mediates ENaC activity via Arp2/3 complex. FASEB J.

[73] Sudarikova AV (2022). Functional role of histamine receptors in the renal cortical collecting duct cells. Am J Physiol Cell Physiol.

[74] Dai XQ (2007). Inhibition of TRPP3 channel by amiloride and analogs. Mol Pharmacol.

[75] Wu MM (2016). Hydrogen peroxide suppresses TRPM4 trafficking to the apical membrane in mouse cortical collecting duct principal cells. Am J Physiol Renal Physiol.

[76] Kanadome T (2019). Systematic screening of depalmitoylating enzymes and evaluation of their activities by the acyl-PEGyl exchange gel-shift (APEGS) assay. Methods Mol Biol.

[77] Kellenberger S (2005). Intracellular thiol-mediated modulation of epithelial sodium channel activity. J Biol Chem.

[78] McDonald FJ (1999). Disruption of the beta subunit of the epithelial Na+ channel in mice: hyperkalemia and neonatal death associated with a pseudohypoaldosteronism phenotype. Proc Natl Acad Sci U S A.

[79] Barker PM (1998). Role of gammaENaC subunit in lung liquid clearance and electrolyte balance in newborn mice. Insights into perinatal adaptation and pseudohypoaldosteronism. J Clin Invest.

[80] Hummler E (1996). Early death due to defective neonatal lung liquid clearance in alpha-ENaC-deficient mice. Nat Genet.

[81] Poulsen SB (2016). Reducing αENaC expression in the kidney connecting tubule induces pseudohypoaldosteronism type 1 symptoms during K+ loading. Am J Physiol Renal Physiol.

[82] Perrier R (2016). Severe salt-losing syndrome and hyperkalemia induced by adult nephron-specific knockout of the epithelial sodium channel α-subunit. J Am Soc Nephrol.

[83] Merillat AM (2009). Conditional gene targeting of the ENaC subunit genes Scnn1b and Scnn1g. Am J Physiol Renal Physiol.

[84] Boscardin E (2017). Severe hyperkalemia is rescued by low-potassium diet in renal βENaC-deficient mice. Pflugers Arch.

[85] Boscardin E (2018). Plasma potassium determines NCC abundance in adult kidney-specific γENaC knockout. J Am Soc Nephrol.

[86] Sorensen MV (2013). Rapid dephosphorylation of the renal sodium chloride cotransporter in response to oral potassium intake in mice. Kidney Int.

[87] Bostanjoglo M (1998). 11Beta-hydroxysteroid dehydrogenase, mineralocorticoid receptor, and thiazide-sensitive Na-Cl cotransporter expression by distal tubules. J Am Soc Nephrol.

