# Supplementary material for: Mice lacking γENaC palmitoylation sites maintain benzamil-sensitive Na+ transport despite reduced channel activity
Source: JCI Insight. 2023 Nov 8;8(21):e172051. doi: 10.1172/jci.insight.172051 (PMC10721255; doi:10.1172/jci.insight.172051)
Supplement: Supplemental data [file jciinsight-8-172051-s273.pdf]

Fig. 1B DNA gel image

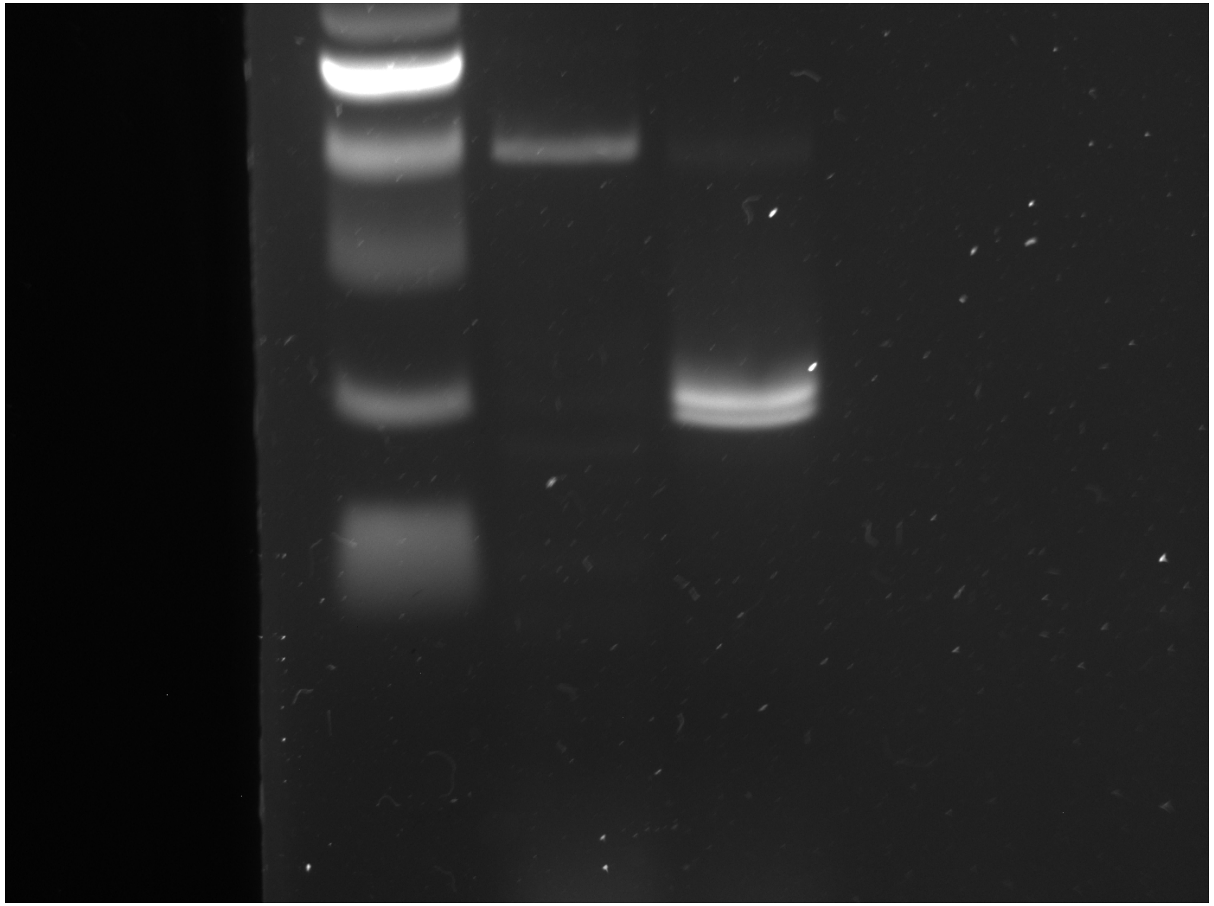

Fig. 3A blots and stain free gel images (males)

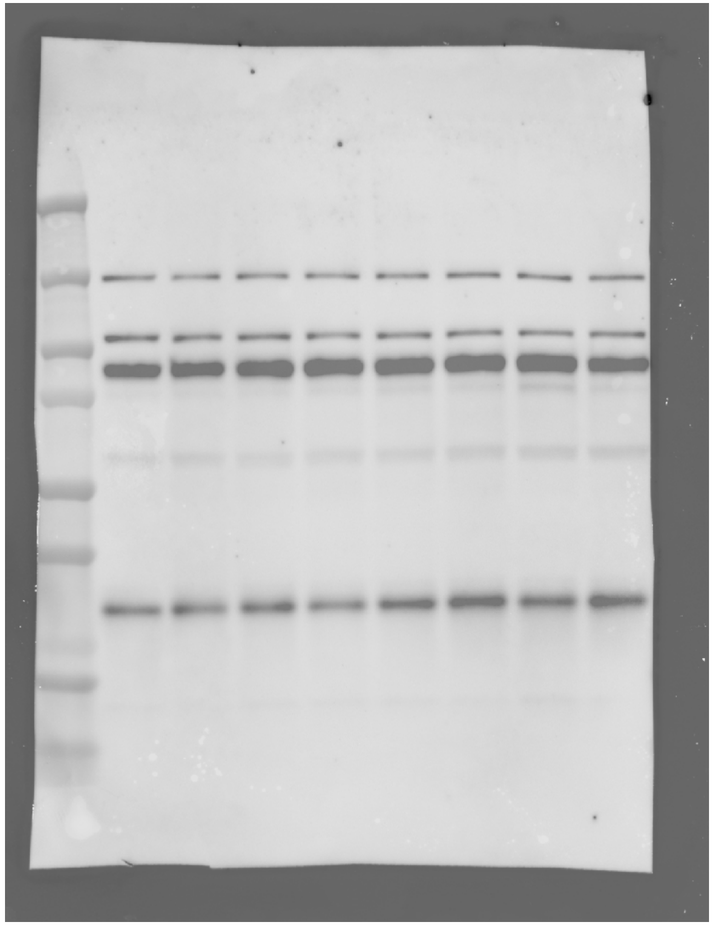

Rabbit anti-α-ENaC
(Loffing group; 1:1000)

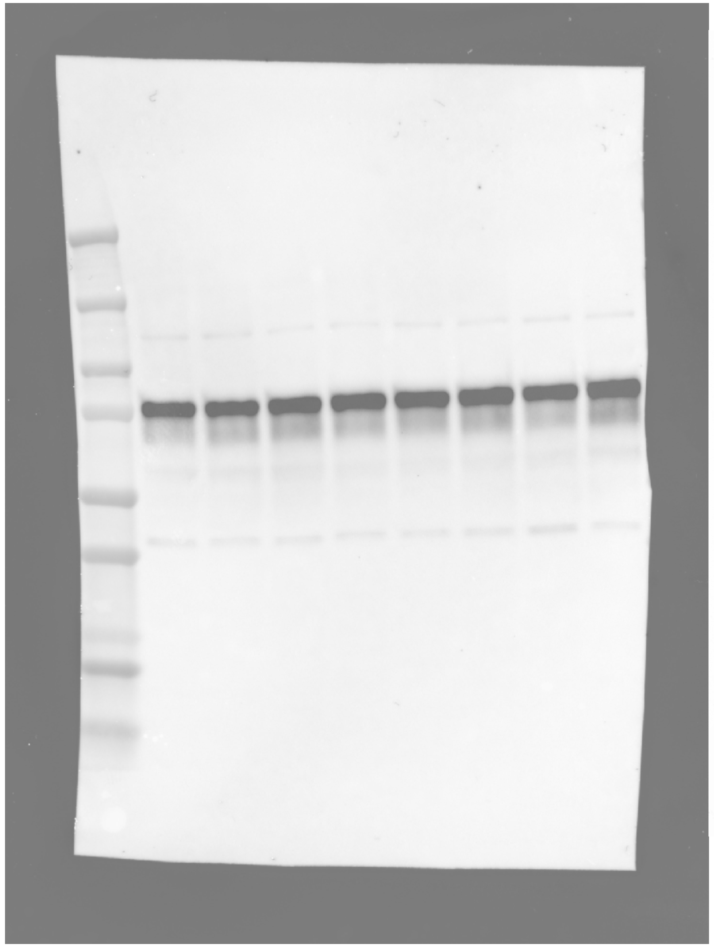

Rabbit anti-γ-ENaC
(Stressmarq; 1:1000)

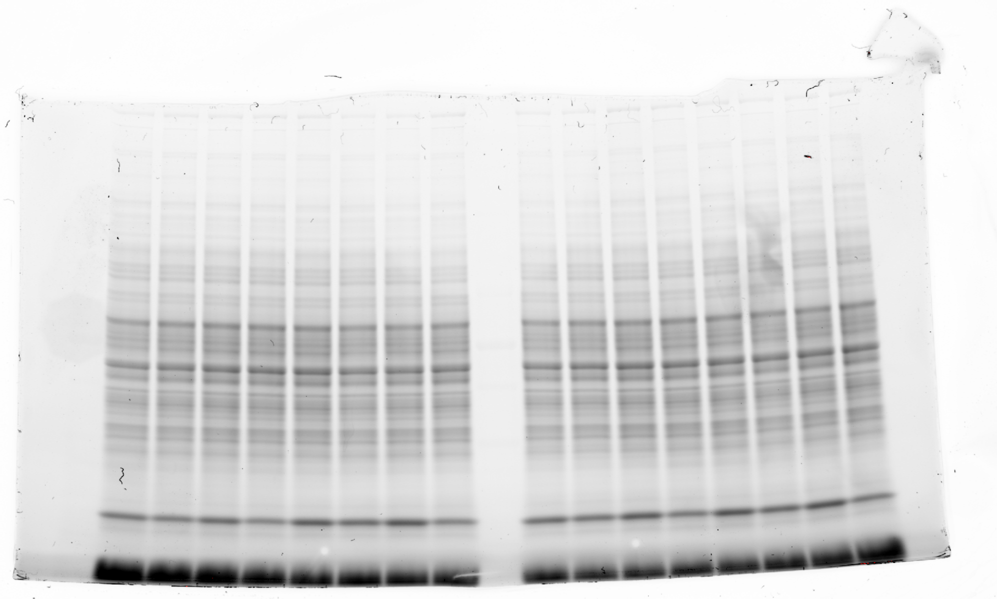

Stain free gel images

Fig. 3B blots and stain free gel images (females)

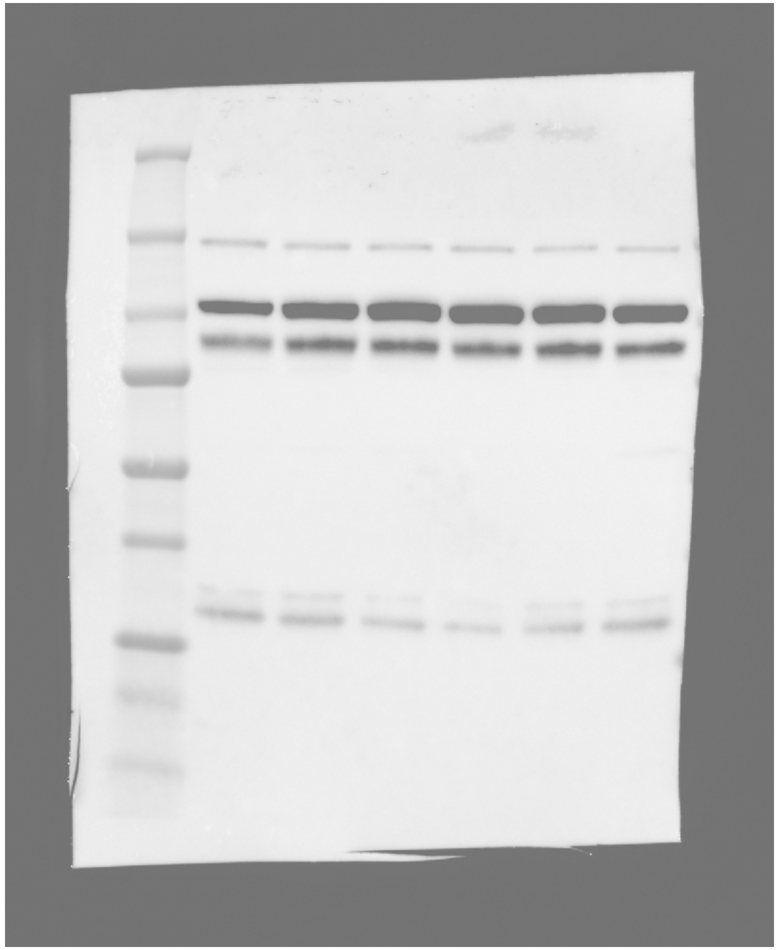

Rabbit anti-α-ENaC
(Loffing group; 1:1000)

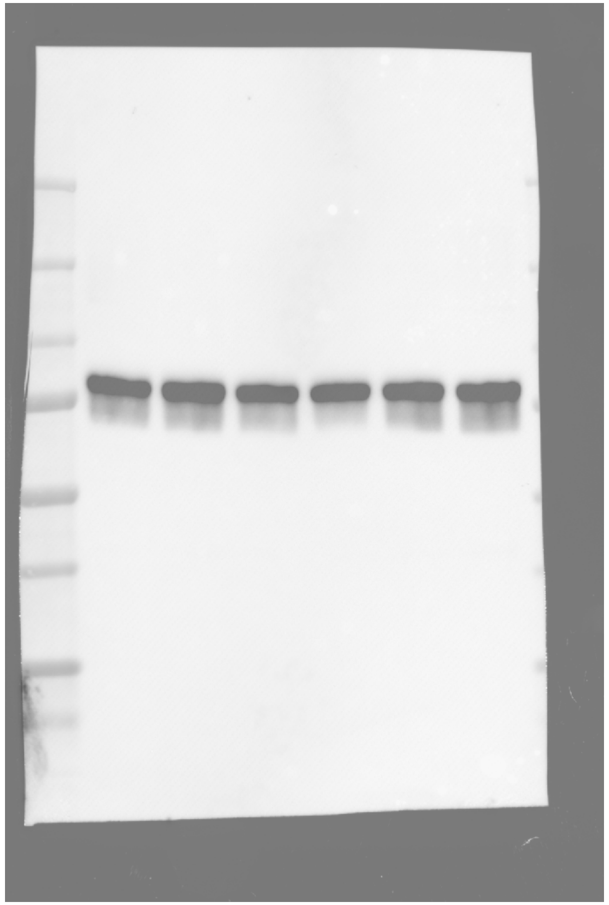

Rabbit anti-γ-ENaC
(Stressmarq; 1:1000)

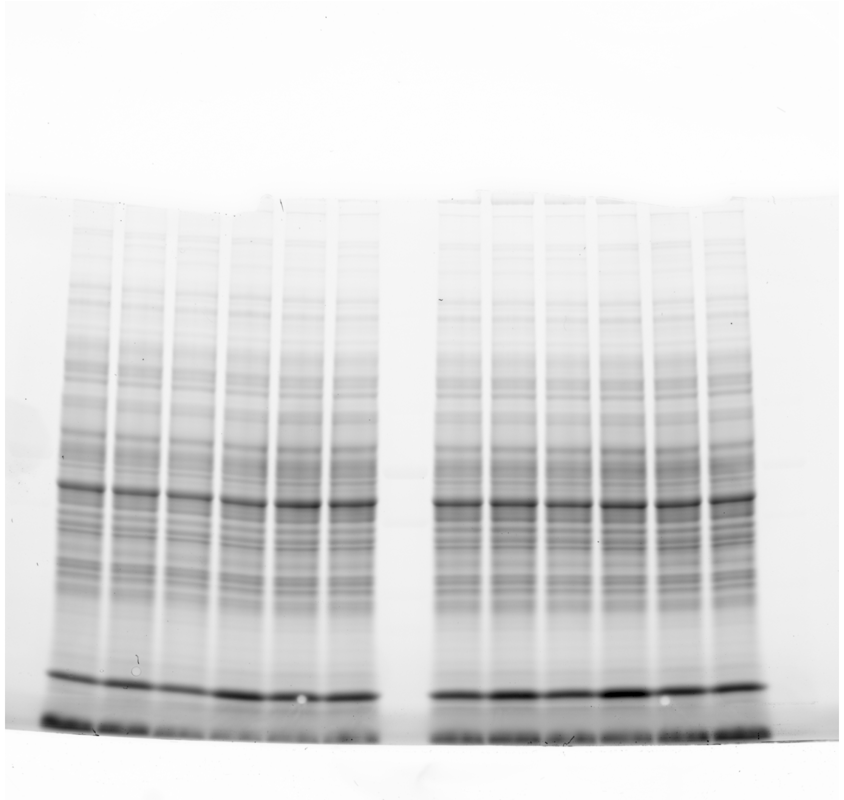

Stain free gel images

Additional samples not shown in Fig. 3, but used for quantification

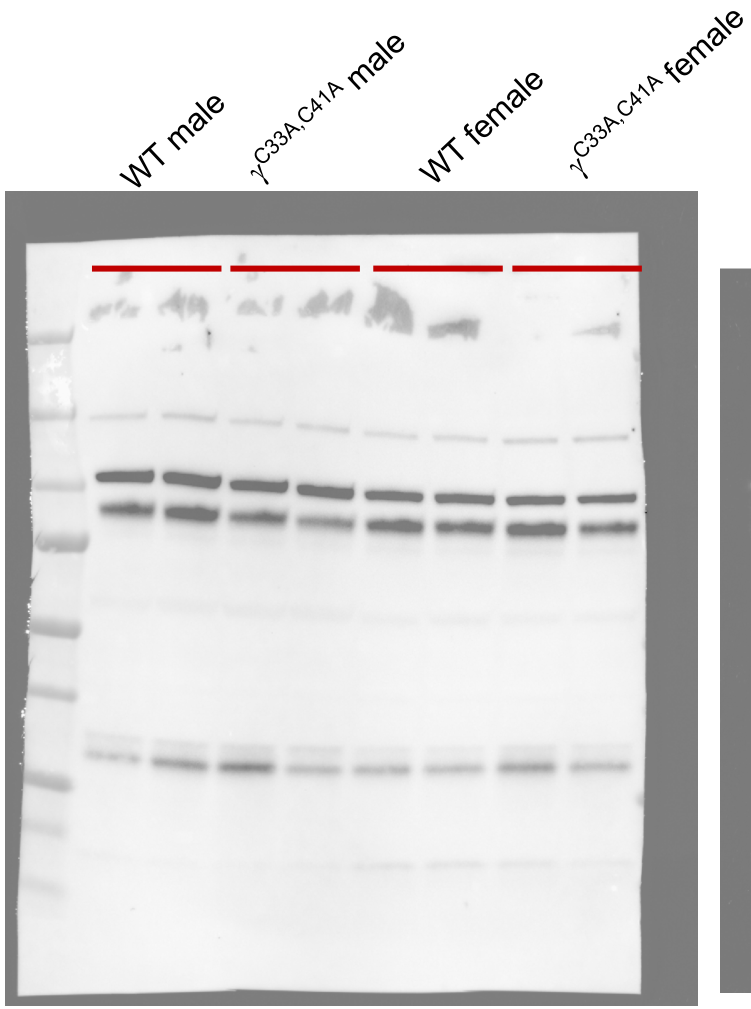

Rabbit anti-α-ENaC
(Loffing group; 1:1000)

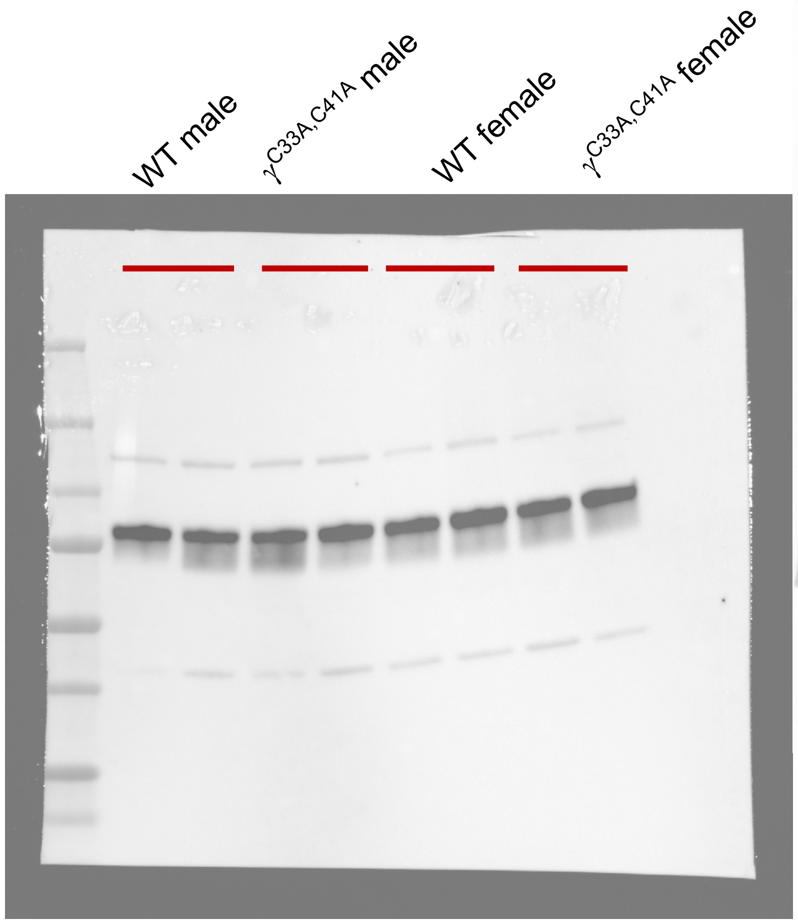

Rabbit anti-γ-ENaC
(Stressmarq; 1:1000)

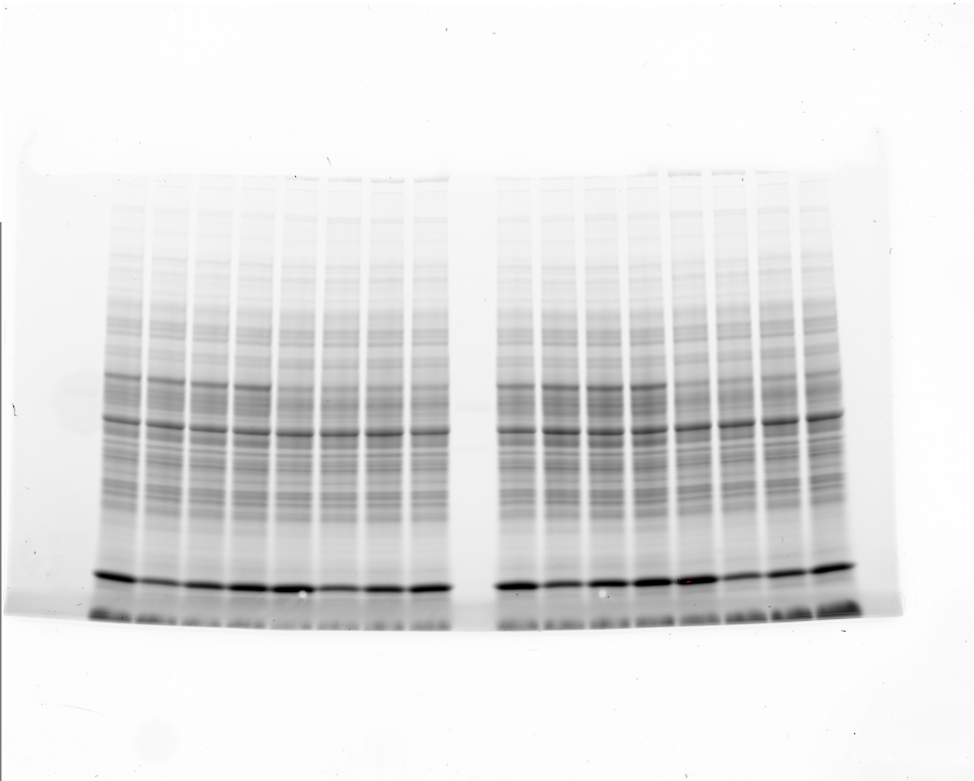

Stain free gel images

Fig. 7 blot and stain free gel images

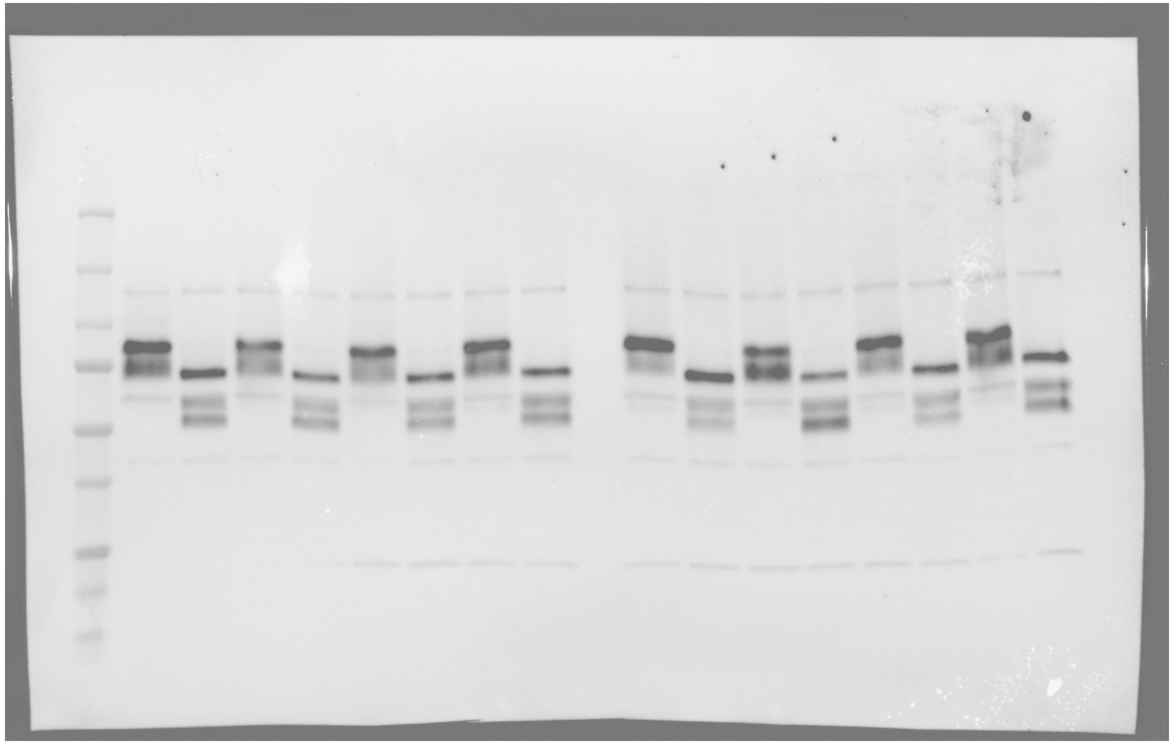

Rabbit anti-γ-ENaC
(Stressmarq; 1:1000)

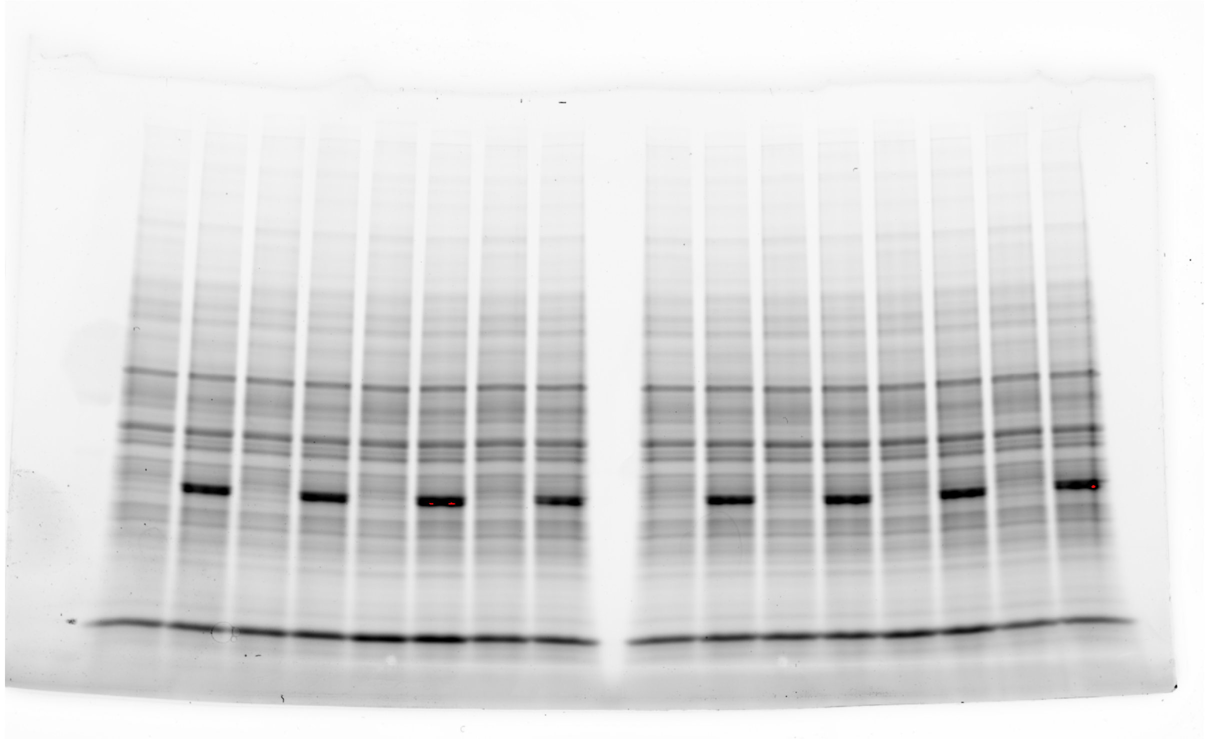

Stain free gel image

Fig. 8 blots and stain free gel images

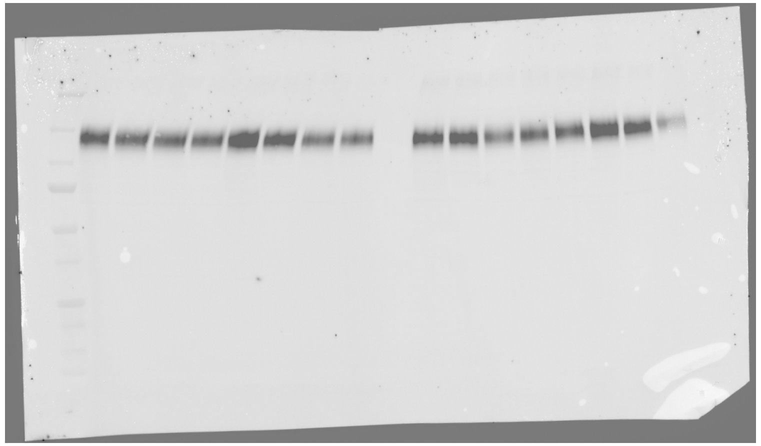

Rabbit anti-phoshoNCC (T53)
(Phosphosolutions; 1:5000)

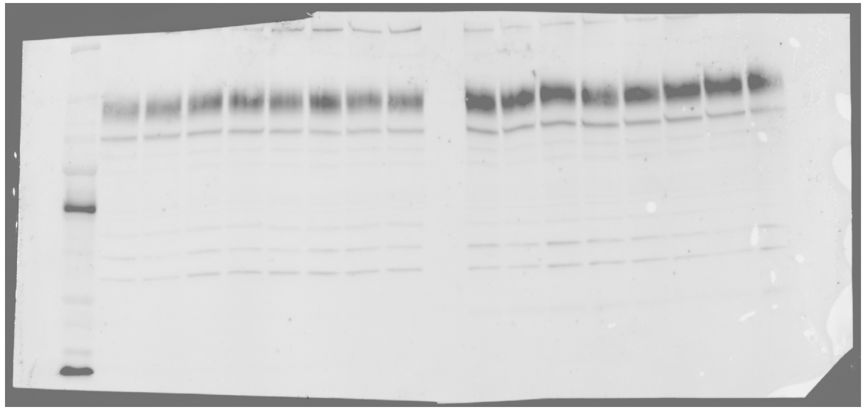

Rabbit anti-NCC
(Ellison lab; 1:5000)

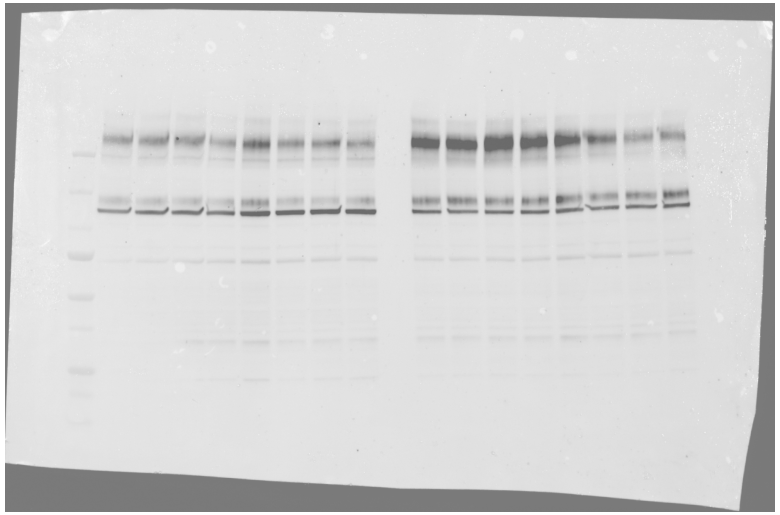

Rabbit anti-NKCC2
(Stressmarq; 1:1000)

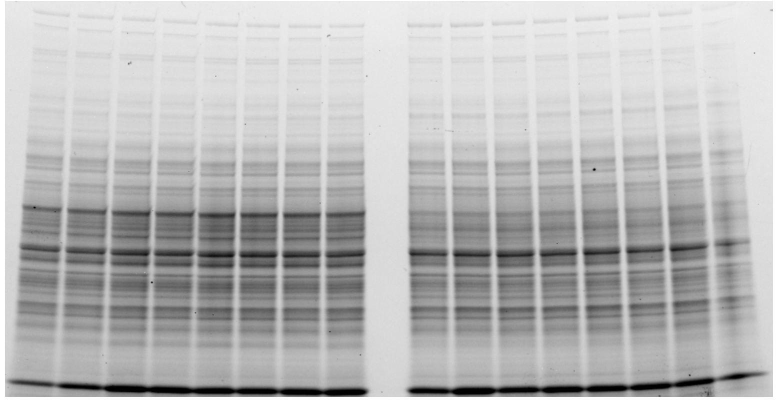

Stain free gel

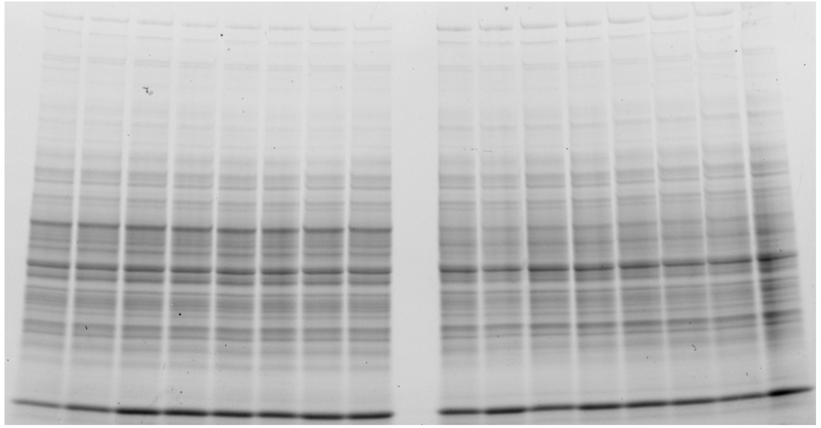

Stain free gel

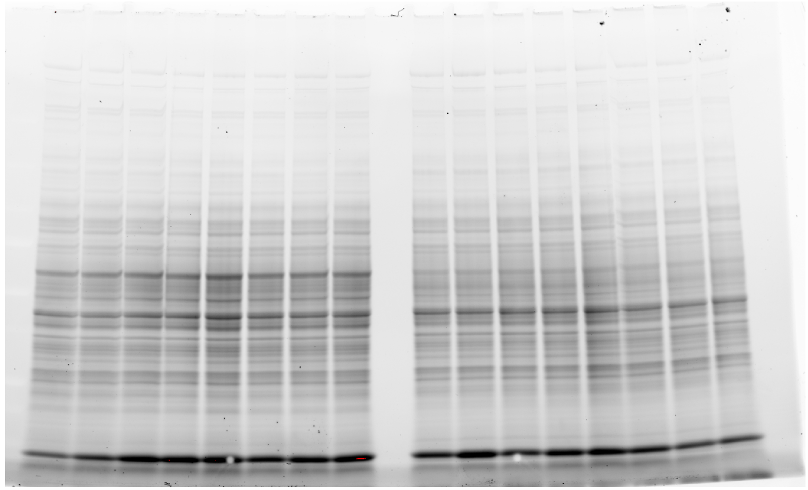

Stain free gel

Fig. 11C blot and stain free gel images

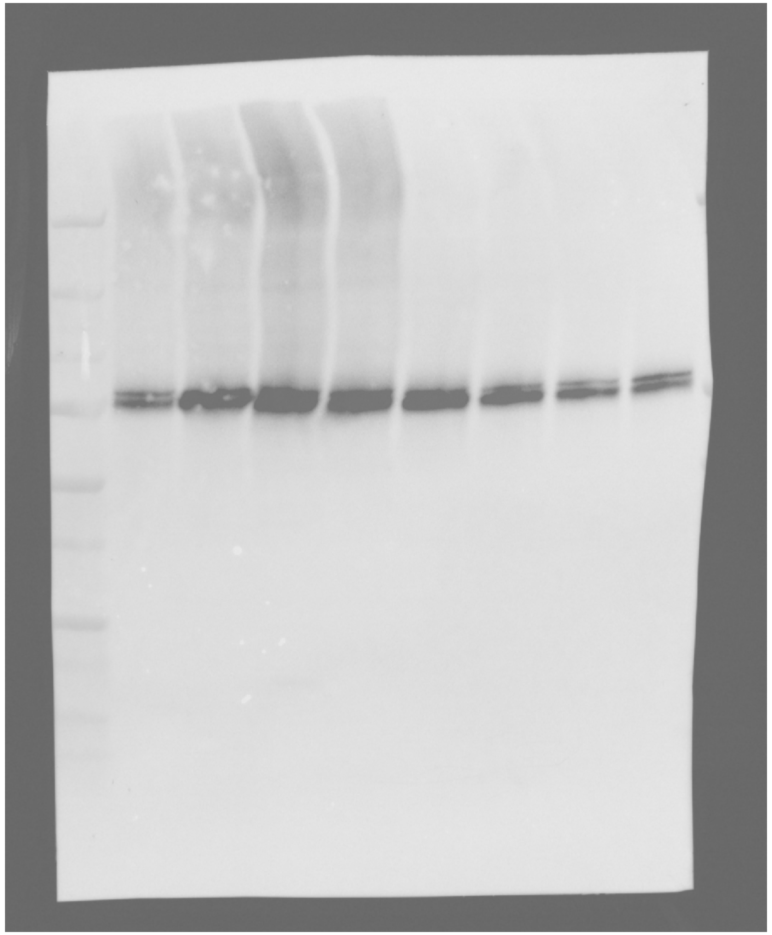

Rabbit anti-γ-ENaC
(Stressmarq; 1:1000)

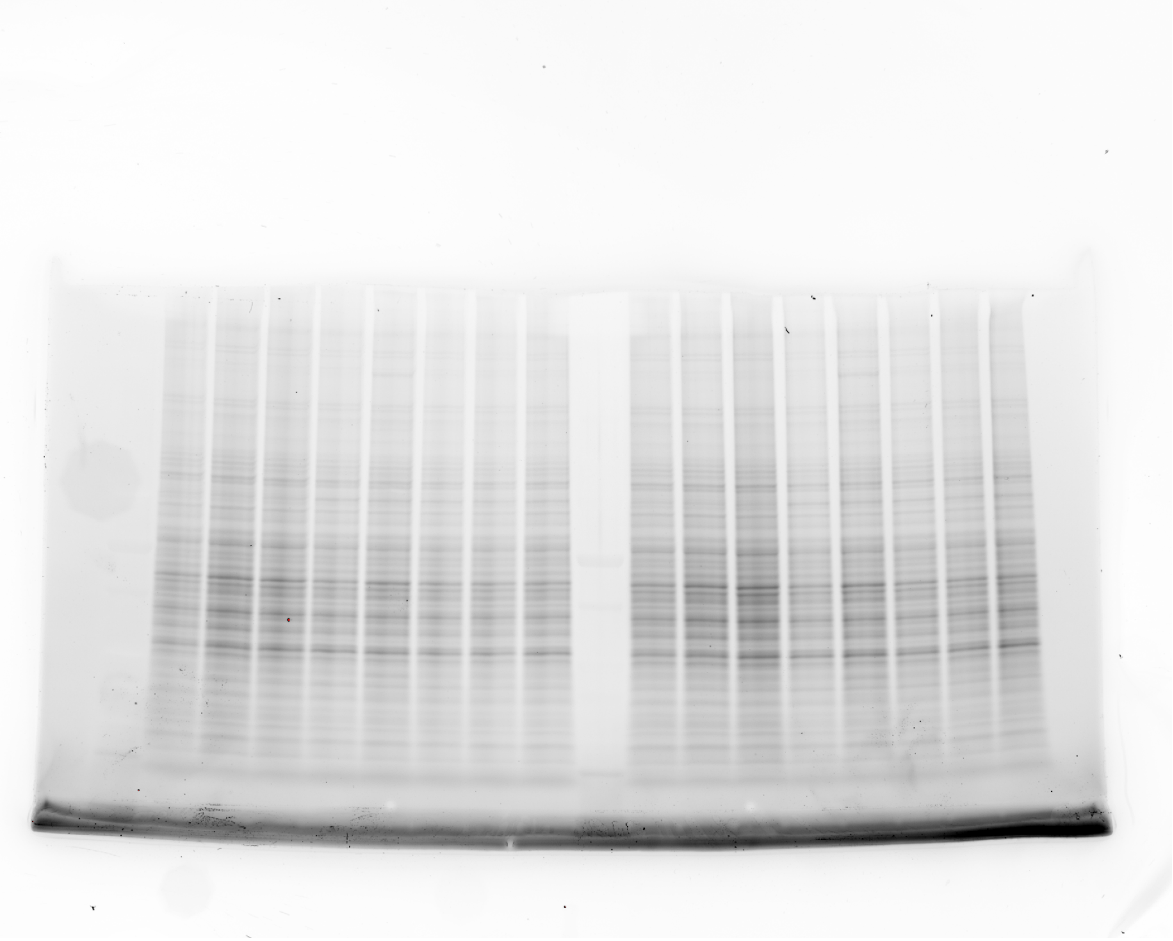

Stain free gel
